# Demyelination Produces a Shift in the Population of Cortical Neurons That Synapse with Callosal Oligodendrocyte Progenitor Cells

**DOI:** 10.1523/ENEURO.0113-25.2025

**Published:** 2025-06-12

**Authors:** Benjamin S. Summers, Catherine A. Blizzard, Raphael R. Ricci, Kimberley A. Pitman, Bowen Dempsey, Simon McMullan, Brad A. Sutherland, Kaylene M. Young, Carlie L. Cullen

**Affiliations:** ^1^Menzies Institute for Medical Research, University of Tasmania, Hobart, Tasmania 7000, Australia; ^2^Tasmanian School of Medicine, University of Tasmania, Hobart, Tasmania 7000, Australia; ^3^Macquarie Medical School, Faculty of Medicine, Health & Human Sciences, Macquarie University, Macquarie Park, New South Wales 2109, Australia; ^4^Mater Research Institute, The University of Queensland, Woolloongabba, Queensland 4102, Australia

**Keywords:** demyelination, NG2 glia, oligodendrocyte progenitor cells, rabies virus, remyelination, synapse

## Abstract

Oligodendrocyte progenitor cells (OPCs) receive synaptic input from a diverse range of neurons in the developing and adult brain. Understanding whether the neuronal populations that synapse with OPCs in the healthy brain is altered by demyelination and/or remyelination may support the advancement of neuroprotective or myelin repair strategies being developed for demyelinating diseases such as multiple sclerosis. To explore this possibility, we employed cre-lox transgenic technology to facilitate the infection of OPCs by a modified rabies virus, enabling the retrograde monosynaptic tracing of neuron→OPC connectivity. In the healthy adult mouse, OPCs in the corpus callosum primarily received synaptic input from ipsilateral cortical neurons. Of the cortical neurons, ∼50% were layer V pyramidal cells. Cuprizone demyelination reduced the total number of labeled neurons. However, the frequency/kinetics of mini-excitatory postsynaptic currents recorded from OPCs appeared preserved. Of particular interest, demyelination increased the number of labeled layer II/III pyramidal neurons and also increased at the expense of layer V pyramidal neurons, a change that was largely ameliorated by remyelination. These data suggest that in the healthy adult mouse brain, callosal OPCs primarily receive synaptic input from cortical layer V pyramidal neurons. However, callosal demyelination is associated with a population switch and OPCs equally synapse with layer II/III and V pyramidal neurons to synapse with OPCs, until myelin is restored.

## Graphical abstract

**Figure:**
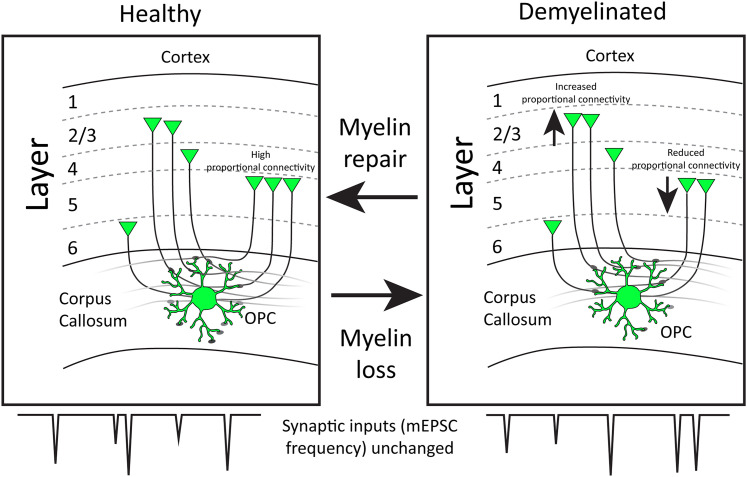
Visual Abstract

## Significance Statement

In the CNS, myelination and remyelination involve the differentiation of oligodendrocyte progenitor cells (OPCs) into new oligodendrocytes (OLs), some of which survive to mature and myelinate axons. Throughout this process, neurons communicate with the OPCs and developing OLs. We show that OPCs in the corpus callosum of adult mice predominantly receive synaptic input from layer V cortical pyramidal neurons. However, they synapse equally with layer II/III and layer V neurons following cuprizone demyelination, suggesting that the highly motile OPC processes select alternative presynaptic sites. Five weeks of remyelination sees OPC connectivity bias return to layer V neurons. This provides critical insight into neuron→OPC communication and cellular interactions that are impacted in demyelinating diseases such as multiple sclerosis.

## Introduction

Oligodendrocyte progenitor cells (OPCs) proliferate to sustain their number and differentiate into myelinating oligodendrocytes (OLs) throughout life (Rivers et al., 2008; Hughes et al., 2013; Young et al., 2013; Hill et al., 2014). In the developing or adult central nervous system (CNS), OPCs receive synaptic input from excitatory (Bergles et al., 2000; Kukley et al., 2007, 2008) and inhibitory neurons (Lin and Bergles, 2004; Balia et al., 2017). Neuron→OPC synapses share key structural, molecular, and physiological characteristics with neuronal synapses, including the presence of postsynaptic scaffolding proteins (Li et al., 2024), neurotransmitter receptors, and voltage-gated ion channels (reviewed in Kula et al., 2019; Xiao and Czopka, 2023) that allow OPCs to detect and respond to neuronal input (Bergles et al., 2000; Lin and Bergles, 2004; Gallo et al., 2008; Kukley et al., 2008; Mangin et al., 2008; Li et al., 2024). While the mechanisms that regulate the assembly and maintenance of neuron→OPC synapses remain largely unknown, they appear to be formed with a degree of selectivity. For example, interneuron→OPC synapses preferentially form with fast-spiking cells (Tanaka et al., 2009; Orduz et al., 2015) and the number of inhibitory inputs decline in early postnatal development (Velez-Fort et al., 2010), such that only a small number of inhibitory connections are retained in adulthood (Moura et al., 2023). Instead, OPCs in the adult mouse brain predominantly synapse with excitatory neurons (Mount et al., 2019; Moura et al., 2023).

Neuron→OPC interactions play an important role in CNS health and disease and are implicated in the regulation of OPC proliferation and differentiation, myelination, and the modulation of axonal and synaptic development and plasticity (see Chen et al., 2018; Habermacher et al., 2019; Fletcher et al., 2021; Moura et al., 2022; Buchanan et al., 2023; Xiao and Czopka, 2023; Hill et al., 2024 for comprehensive reviews). In the developing CNS, electrically active axons are preferentially myelinated (Hines et al., 2015) and the chemogenetic activation of CTIP2^+^ neurons in the adult mouse hippocampus similarly increases synapse formation between CTIP2^+^ neurons and OPCs (Moura et al., 2023). Neuron→OPC synapses also appear to support remyelination, as OPCs rapidly populate demyelinated lesions and synaptically integrate prior to differentiation (Etxeberria et al., 2010; Gautier et al., 2015; Sahel et al., 2015). This process is at least partially activity dependent, as blocking neuronal neurotransmitter release reduces OPC differentiation and lesion remyelination (Gautier et al., 2015). As OPCs form de novo synapses with demyelinated axons (Gautier et al., 2015), it is likely that they receive synaptic input from distinct or at least different neuronal populations in the healthy versus demyelinated brain.

The corpus callosum is the primary commissural region of the brain, composed of myelinated and unmyelinated axons (Sturrock, 1980; Lamantia and Rakic, 1990; Reeves et al., 2012), largely originating from layer II/III and V excitatory cortical pyramidal neurons that span the two hemispheres of the cerebral cortex (Chovsepian et al., 2017; Ku and Torii, 2020). OPCs receive synaptic input from these axons (Kukley et al., 2007; Ziskin et al., 2007; Mount et al., 2019), and the myelination of callosal axons continues throughout life (Young et al., 2013; Tripathi et al., 2017). To determine whether OPCs receive synaptic inputs from discrete subsets of projection neurons, we conditionally infected a small number of OPCs in the subcortical white matter with a monosynaptically restricted, glycoprotein-deleted rabies virus (Wall et al., 2010; Mount et al., 2019; Moura et al., 2023). This approach resulted in the transsynaptic labeling of cortical neurons that project their axons through the corpus callosum, alongside hippocampal neurons that project their axons through the alveus. In healthy adult mice, OPCs received synaptic connections from excitatory neurons in cortical and subcortical regions, largely located within the same hemisphere. Following cuprizone-induced demyelination, the total number of neurons that synapsed onto an OPC was reduced and a higher proportion of the synaptic inputs came from layer II/III cortical pyramidal neurons, at the expense of layer V pyramidal cells. After remyelination, the distribution of virally labeled neurons was partially restored to that observed in the healthy brain. These data suggest that callosal OPCs preferentially receive synaptic input from layer V excitatory cortical neurons, but that demyelination removes this preference, and that layer II/III and layer V neurons are equally likely to synapse with OPCs.

## Materials and Methods

### Pseudotyped glycoprotein-deleted rabies virus

A glycoprotein-deleted rabies virus encoding EGFP (SADΔG-GFP) was amplified and pseudotyped with the envelope protein of the avian sarcoma leukosis virus subtype A (EnvA) to produce SADΔG-(EnvA)-GFP as previously described (Osakada and Callaway, 2013; Dempsey et al., 2017). Briefly, B7GG cells were cultured in Dulbecco's modified eagle medium plus GlutaMAX (DMEM; Invitrogen)/10% fetal calf serum (FCS; Invitrogen, 10099-141) at 37°C and 5% CO_2_. At ∼60% confluency, the B7GG cells were washed with PBS and exposed to DMEM/10% FCS/40% SADΔG-GFP supernatant (from a previous culture) and maintained at 35°C and 3% CO_2_ for 5–6 h to enable infection. The medium was replaced with DMEM/10% FCS and the cells cultured at 35°C/3% CO_2_ to support SADΔG-GFP replication and release into the culture medium. Supernatant containing the unpseudotyped virus was filtered (0.22 µm; Millipore, SCGP0525) and stored at −80°C.

To pseudotype the virus, BHK-EnvA cells were cultured in DMEM/10% FCS at 35°C/3% CO_2_. When ∼60% confluent, the cells were cultured in DMEM/10% FCS/50% unpseudotyped SADΔG-GFP supernatant for 5–6 h. The cells were washed, passaged, and replated at 20% confluency in DMEM/10% FCS and cultured at 35°C/3% CO_2_ for ∼48 h. The pseudotyped SADΔG-GFP-(EnvA) viral supernatant was collected and filtered, and the SADΔG-GFP-(EnvA) virus concentrated by ultracentrifugation at 24,000 rpm (∼72,000 × *g*) for 2 h at 4°C using a Sorvall WX+ Ultracentrifuge (Thermo Fisher Scientific, 75000100) with a TH-641 Swinging Bucket Rotor (Thermo Fisher Scientific, 54295). The viral pellet was resuspended in 300 µl Hanks’ balanced salt solution (HBSS; Invitrogen, 14185052) per tube, then pooled and added to the surface of a 20% sucrose (Sigma)/HBSS cushion (2 ml), and topped with HBSS, before centrifugation at 20,000 rpm (∼51,000 × *g*) for 2.5 h at 4°C. The supernatant was aspirated, and the SADΔG-GFP-(EnvA) viral pellet gently resuspended in 100 μl HBSS and placed at ∼21°C for 1 h. Aliquots of concentrated SADΔG-GFP-(EnvA) were stored at −80°C for experimental use.

Effective pseudotyping was confirmed by inoculating HEK293T cells or HEK293T-TVA800 cells (constitutively express the receptor for EnvA), with SADΔG-(EnvA)-GFP. Cells were plated in 24-well plates (Corning, CLS3524) at a density of 1.5 × 10^5^ and cultured in DMEM/10% FCS at 35°C/3% CO_2_ before viral inoculation in serial dilutions (10^3^–0^9^ in DMEM/10% FCS). The number of GFP^+^ cells per well was quantified 36 h postinoculation. Viral titer was calculated using the following equations:

$$\mathrm{Titer}\;\mathrm{of}\;\mathrm{viral}\;\mathrm{stock}\;=\;(\mathrm{average}\;\mathrm{number}\;\mathrm{of}\;\mathrm{GF}P^{+\;}\mathrm{cells}\;\mathrm{per}\;\mathrm{well}/0.75\;\times \;3)\times \mathrm{viral}\;\mathrm{preparation}\;\mathrm{dilution}\;\mathrm{factor}.$$

Final SADΔG-(EnvA)-GFP viral titers were 2.17 × 10^6^–3.06 × 10^6^ infectious units/ml. The absence of GFP fluorescence in inoculated HEK293T cells confirmed that the pseudotyped SADΔG-GFP-(EnvA) virus could only infect TVA receptor^+^ cells.

### Transgenic mice

Animal experiments were approved by the University of Tasmania Animal Ethics Committee (A0013741 and A0016151) and carried out in accordance with the Australian code of practice for the care and use of animals for scientific purposes. All mice were weaned at postnatal day (P) 35 and group housed (2–5 per cage) in individually ventilated Optimice microisolator cages (Animal Care Systems) at 21 ± 2°C on a 12 h light/dark cycle (lights on 07:00 to 19:00). Mice had *ad libitum* access to standard rodent chow (Barastoc rat and mouse pellets) and water.

Homozygous *RphiGT* transgenic mice [*B6;129P2-Gt(ROSA)26Sor^tm1(CAG-RABVgp4,-TVA)Arenk/J^*, RRID:IMSR_JAX:024708; Takatoh et al., 2013] that, following Cre-mediated recombination, express the TVA receptor and rabies glycoprotein under the control of the *Rosa26-YFP* promoter, and heterozygous *Pdgfrα-H2BGFP* transgenic mice (RRID:IMSR_JAX:007669; Hamilton et al., 2003), were purchased from Jackson Laboratories. *Pdgfrα-CreER^T2^* (Rivers et al., 2008) transgenic mice were a kind gift from Prof. William D Richardson (University College London). All mice were backcrossed and maintained on a *C57BL6/J* background. Heterozygous *Pdgfrα-CreER^T2^* mice were crossed with homozygous *RphiGT* mice to produce *Pdgfrα-CreER^T2^:: RphiGT* experimental mice. Male and female mice were randomly assigned to treatment groups, but care was taken to ensure that littermates were represented across treatment groups.

*Pdgfrα-H2BGFP* and *Pdgfrα-CreER^T2^* mice were genotyped as described (Ferreira et al., 2020; Zhen et al., 2022). In brief, genomic DNA (gDNA) was extracted from ear biopsies by ethanol precipitation and PCR performed using 50–100 ng of gDNA with the following primer combinations: *Cre 5′* CAGGT CTCAG GAGCT ATGTC CAATT TACTG ACCGTA and Cre 3′ GGTGT TATAAG CAATCC CCAGAA; *GFP 5′* CCCTG AAGTTC ATCTG CACCAC and *GFP 3′* TTCTC GTTGG GGTCT TTGCTC. *RphiGT* mice were genotyped by PCR detection of TVA using the following primers: *TVA 5*′ CGGTT GTTGG AGCTG CTGGT GCTGC TGC and *TVA 3*′ TGCAA CGATC AGCAT CCACA TGC. The PCR program (94°C for 4 min, followed by 35 amplification cycles of 94°C for 30 s, 62°C for 45 s, and 72°C for 60 s, and 10 min at 72°C) produced DNA products of ∼500 bp for Cre, ∼242 bp for GFP, and ∼300 bp for TVA.

### Tamoxifen administration

Tamoxifen (Sigma; 10540-29-1) was dissolved to 40 mg/ml in corn oil (Sigma; 8001-30-7) by sonication at 21°C for ∼2 h and administered to adult mice by oral gavage as four consecutive daily doses (300 mg/kg). Mice received four consecutive doses of tamoxifen, as we have previously shown that this dosing regimen produces maximum activation of Cre recombinase in OPCs in Pdgfra-CreER^T2^ mice (Rivers et al., 2008). We also ensured a minimum of 3 d between the final tamoxifen dose and commencing any intervention, to ensure that OPC recombination was complete and stable prior to viral injection (Rivers et al., 2008). We avoided the simultaneous delivery of tamoxifen and cuprizone, as this can result in weight loss, but primarily because tamoxifen can promote remyelination (Gonzalez et al., 2016). To evaluate neuron-OPC synaptic connectivity in remyelinating mice, tamoxifen delivery was delayed until the end of the remyelinating period, to ensure that essentially all callosal OPCs, including those generated from neural stem cells over the 10 week tracing period (Xing et al., 2014), were targeted by recombination.

### Cuprizone-induced demyelination and remyelination

To induce demyelination, mice received a diet containing 0.2% (w/w) cuprizone (bis-cyclohexanoneoxaldihydrazone; Sigma, C9012) from P49 for five consecutive weeks. Cuprizone was thoroughly mixed with dry, ground standard rodent chow (Barastoc rat and mouse pellets) and rehydrated with water in a 3:1 (w/v) ratio (Nguyen et al., 2024). Cuprizone chow was prepared as large experimental batches and distributed across cages so that each mouse had *ad libitum* access to ∼25 g of hydrated cuprizone-chow, replaced every 2 d.

### Stereotaxic viral microinjections

Adult mice received a subcutaneous (s.c.) injection of the nonsteroidal anti-inflammatory analgesic meloxicam (Metacam 5 mg/kg at 0.0125%; Ilium, Troy Laboratories) prior to anesthesia induction with 5% isoflurane (Henry Schein) in O_2_ delivered at ∼0.6 L per minute (Darvall Isoflurane Precision Vapouriser; GVPAD- 09175S). Anesthesia was maintained with 1.2–2% isoflurane in O_2_ throughout the procedure. Anesthetized mice were secured within a stereotaxic frame (Narishige, SR-5R-HT) and Viscotears liquid eye gel (Novartis Pharmaceuticals) applied to maintain eye moisture. Following sterilization with Microshield chlorhexidine (Schulke), a cocktail of bupivacaine hydrochloride (1 mg/kg at 0.025%; Pfizer) and lignocaine hydrochloride (5 mg/kg at 0.03%; Mavlab) was administered subcutaneously to the scalp to induce local analgesia and 0.9% sterile saline (Livingstone International, DWSC0005) administered to the left or right flank (0.5 ml, s.c.) to prevent dehydration during the procedure. Mice were placed on sterile heating pads to maintain body temperature at 37°C, and the reflex response, breathing rate, mucous membrane color, and capillary refill time (MM/CRT) periodically monitored throughout the surgical procedure. A small cranial incision (≤1 cm) was made in the scalp to expose the skull and the periosteum removed. A small burr hole (0.5 mm tip diameter burr) was microdrilled (Fine Science Tools, 19007-05) through the right side of the skull, 1.5 mm posterior and 1 mm lateral to bregma, ensuring the dura remained intact. Then, 800 nl of SADΔG GFP-(EnvA) was injected into the corpus callosum (∼1 mm below the dura) at a rate of 100nl/min using a UMP3 UltraMicroPump (World Precision Instruments) attached to a 5 μl Hamilton Neuros Syringe (model 75 RN) with 33 gauge beveled needle (Hamilton, 65460-03). The needle was slowly withdrawn to ensure fluid diffusion, and the surgical incision was sutured (Daclon Nylon, GVPI-915 1512). Mice were recovered in their home cage on a 37°C heat pad for ∼30 mins until awake, eating and grooming normally.

### Electrophysiology

Adult *Pdgfrα-H2BGFP* mice were killed by cervical dislocation and the brains rapidly dissected into ice-cold sucrose solution (in mM: 75 sucrose, 87 NaCl, 2.5 KCl, 1.25 NaH_2_PO_4_, 25 NaHCO_3_, 7 MgCl_2_, and 0.95 CaCl_2_). Then, 250 μm acute coronal brain slices were generated using a Leica VT1200s Vibratome and incubated for 30 min at ∼32°C in artificial cerebral spinal fluid (300 ± 5 mOsm/kg ACSF; in mM: 119 NaCl, 1.6 KCl, 1 NaH_2_PO_4_, 26.2 NaHCO_3_, 1.4 MgCl_2_, 2.4 CaCl_2_, and 11 glucose) saturated with 95% O_2_/5% CO_2_ and maintained at ∼21°C in ACSF saturated with 95% O_2_/5% CO_2_.

Whole-cell patch-clamp recordings were made from GFP^+^ callosal OPCs in the body of the corpus callosum, underlying the parietal/retrosplenial cortices; the area corresponding to the location of viral injection for the neuroanatomical studies (∼bregma 1.5 mm). Recording pipettes, pulled from glass capillaries had a resistance of 3.6–6.9 MΩ (Harvard Apparatus, W3 30-0057), were filled with a cesium-based internal solution containing the following (in mM): 125 Cs-methanesulfonate, 4 NaCl, 3 KCl, 1 MgCl_2_, 8 HEPES, 9 EGTA, 10 phosphocreatine, 5 MgATP, and 1 Na_2_GTP set to a pH of 7.2 with CsOH, with an osmolarity of 290 ± 5 mOsm/kg (Fulton et al., 2010). Upon breakthrough, access resistance (Ra), capacitance (Cm), and membrane resistance (Rm) were recorded using a HEKA patch-clamp EPC800 amplifier and pClamp 10.5 software (Molecular Devices). As OPCs produce a transient inward current after depolarization beyond −30 mV, the voltage-gated inward current was evaluated for each GFP^+^ cell by recording their response to 500 ms voltage steps of −70 to −20 mV, −10, 0, +10, and +20 mV. Cells with a capacitance (Cm) of <50 pF and NaV conductance ≥100 pA for the −70 to 0 mV step were classified as OPCs (Pitman et al., 2020). Cells were held at −70 mV and mEPSCs recorded from slices perfused in ACSF containing 1 μM tetrodotoxin (TTX; Abcam), as 2 × 3 min gap-free protocols, sampled at 50 kHz and filtered at 3 Hz (total of 6 min recording per cell). Perfusate was then replaced with ACSF containing 1 μM TTX and 100 μM of the secretagogue ruthenium red (Sigma), and after ≥3 min perfusion, the recording protocol was repeated. Ra was measured before and after each recording, and a cell was excluded if the Ra exceeded 20 MΩ or deviated >20% from the baseline Ra measurement. As OPCs have a high Rm (>1 GΩ), recordings were made without series resistance compensation. Data have not been adjusted for the liquid junction potential. Measurements were made from data files using Clampfit 10.5 (Molecular Devices) and mEPSCs with an amplitude ≥5 pA were identified and analyzed using the MiniAnalysis60 program (Synaptosoft).

### Tissue preparation and immunohistochemistry

Mice were terminally anesthetized by an intraperitoneal (i.p.) injection of 300 mg/kg sodium pentobarbital (Ilium) prior to transcardial perfusion with 4% (w/v) paraformaldehyde (PFA; Sigma) in PBS. Brains were dissected and immersion fixed in 4% PFA for 90 min at ∼21°C. SADΔG-GFP-(EnvA) transfected brains were transferred to 4°C PBS and the olfactory bulbs and cerebellum removed prior to embedding in 4% (w/v) molecular grade agarose (Bioline Australia) in PBS. Floating serial coronal vibratome sections (40 μm; Leica VT1000S Vibratome) were used for immunohistochemistry (see below), mounted on glass slides (Dako, K8020), and coverslipped with fluorescent mounting medium (Dako). To detect myelin basic protein (MBP), perfusion fixed brains were instead sliced into 2 mm coronal slices using a 1 mm brain matrix (Kent Scientific) and further immersion fixed in 4% PFA for 90 min at ∼21°C. The brain slices were cryoprotected in 20% (w/v) sucrose (Sigma)/PBS in 4°C overnight and frozen in Shandon Cryomatrix (Thermo Fisher Scientific) using liquid nitrogen. Brain slices were stored at −80°C until coronal brain cryosections (30 μm, ∼bregma −1.5 mm) were floated onto glass slides, allowed to dry, and exposed to −20°C methanol for 10 min prior to commencing immunohistochemistry.

For immunohistochemistry, vibratome sections or cryosections were incubated in blocking solution [0.1% (v/v) Triton X-100 (Sigma) and 10% FCS in PBS] for 1 h at ∼21°C. All antibodies were diluted in blocking solution, applied to sections, and incubated overnight at 4°C. Primary antibodies included the following: goat anti-PDGFRα (1:200; R&D Systems, RRID: AB_2236897), rat anti-GFP (1:2,000; Nacalai Tesque, RRID: AB_10013361), rat anti-MBP (1:200; Abcam, RRID: AB_305869), rabbit anti-ASPA (1:200; Millipore, RRID: AB_2827931), mouse anti-NeuN (1:500; Millipore, RRID: AB_2298772), rabbit anti-parvalbumin (1:2,000; Abcam, RRID: AB_2924658), and rabbit anti-GFAP (1:1,000; Dako, RRID: AB_10013382). Secondary antibodies were conjugated to AlexaFluor-488, −568 or −647 (Invitrogen) and included the following: donkey anti-mouse (1:1,000), donkey anti-goat (1:1,000), donkey anti-rabbit (1:1,000), and donkey anti-rat (1:500). Nuclei were labeled using Hoechst 33342 nuclear stain (1:1,000; Invitrogen, 62249).

### Microscopy and image analysis

Low (20× air objective) and high (40× water objective) magnification confocal images were collected using an UltraView Nikon Ti Confocal Microscope with Volocity Software (PerkinElmer), or Ultraview Eclipse Nikon Ti Microscope with a differential spinning disk attachment (ANDOR) running NIS-Elements Advanced Research software (Nikon). Standard excitation and emission filters for DAPI (Hoechst 33342), FITC (Alexa Fluor-488), TRITC (Alexa Fluor-568), and CY5 (Alexa Fluor-647) were used. Images were captured with a 1–3 μm *z*-spacing and stitched to form a composite image of a defined region of interest.

To quantify myelin content, a 20× single *z*-plane image was collected for a single field of view of the medial corpus callosum for each coronal cryosection immunolabeled to detect MBP. Images were analyzed in ImageJ (NIH) by a researcher blind to the experimental conditions. Images were changed to 16 bit grayscale and manual thresholding performed to identify all MBP^+^ pixels within the dorsal fornix—a white matter tract largely unaffected by cuprizone feeding (Yang et al., 2009; Hertanu et al., 2023). MBP coverage was then quantified in the corpus callosum, using the same thresholding parameter. The proportion of the *x*–*y* area covered by MBP labeling was quantified in three randomly placed 100 μm^2^ boxes in the medial corpus callosum for each of three sections per mouse, and data are expressed as mean area covered by MBP^+^ pixels (%).

To quantify the number and regional distribution of RABV-GFP^+^ cells, 20× confocal image stacks (3 μm *z*-spacing) were captured from serial coronal sections at and surrounding the injection site and were stitched to form composite images. Manual cell counts were performed using Photoshop CS6 (Adobe) by an experimenter blind to treatment. Each brain region was defined with reference to the Hoechst nuclear staining pattern and anatomical features cross-referenced against the Mouse Brain Atlas (Franklin and Paxinos, 2007). RABV-GFP^+^ cells were included in the analysis if they contained a Hoechst^+^ nucleus and a cellular identity assigned if the cell coexpressed cell-specific markers and/or could be morphologically categorized. More specifically, RABV-GFP^+^ cells that expressed PDGFRα were identified as OPCs, while those that expressed ASPA had a small spherical nucleus and supported multiple straight fluorescent segments characteristic of myelin sheaths (Osanai et al., 2017) were identified as OLs. Astrocytes were identified by their expression of glial fibrillary acidic protein (GFAP) or morphologically distinct star-shaped cell bodies and highly ramified processes (Matyash and Kettenmann, 2010; Vasile et al., 2017; Zhou et al., 2019). Neuronal nuclear protein (NeuN) or parvalbumin (PV) expression were used to help classify RABV-GFP^+^ neurons. Excitatory pyramidal neurons were further classified by their distinct pyramidal shaped cell bodies and apical and basal dendritic trees including their large apical dendrites with a high density of dendritic spines (De Felipe and Fariñas, 1992). Interneurons colabeled for parvalbumin and/or elaborated a smaller dendritic tree that lacked spines and could be identified with reference to previously defined interneuron classifications (Porter et al., 2001; Dumitriu et al., 2007; Feldmeyer et al., 2018; Laturnus et al., 2020).

The number and anatomical distribution of RABV-GFP^+^ neurons in each brain was quantified by performing high-throughput imaging of serial coronal vibratome brain sections using an Olympus VS120 Slide Scanner (Olympus) fluorescent microscope, running VS200 ASW V3.3 software, with standard excitation and emission filters for DAPI (Hoechst 33342), FITC (Alexa Fluor-488), and TRITC (Alexa Fluor-568). Low magnification (10× air objective) images were captured at 5 μm *z*-spacing across the central 35 μm of each section and stitched to form a single composite maximum projection image of each coronal brain section. Images were converted to 16 bit RGB TIFF files before blinded cell counts were performed manually in Photoshop CS6. Brain regions and cortical laminar were defined using Hoechst nuclear staining and anatomical landmarks defined in the Mouse Brain Atlas (Franklin and Paxinos, 2007).

RABV-GFP^+^ cells that possessed a clear Hoechst-labeled nucleus were quantified across ∼120 sequential serial sections per brain between ∼bregma 2.0 and −4.0, with an estimated ≤2% of tissue lost during tissue sectioning. Sections containing the remaining rostral portion of the cortex and cerebellum were analyzed in a small number of mice, but as no RABV-GFP^+^ neurons were identified within these regions, they were excluded from future analyses. To account for variability in the number of RABV-GFP^+^ neurons/glia labeled within each brain, data were normalized by expressing the number of RABV-GFP^+^ cells in each region as a percentage of the total RABV-GFP^+^ cells detected in each brain or within a defined anatomical region.

To quantify cFos expression by NeuN^+^ neurons in the cortex, previously generated confocal image stacks that were stitched to encompass the visual cortex of *n* = 3 control and 5 week 0.2% (w/w) cuprizone demyelinated mice (Nguyen et al., 2024) were downloaded for analysis. Each image captured Hoescht33342, NeuN, and c-fos labeling. Cortical laminar were defined based from nuclei distributions using Hoescht33342. Cell quantification was performed manually in layers II/III and V. Each NeuN^+^ cell was identified and counted in layer II/III or V of the visual cortex using the cell counter function in Image J (NIH). These data were used to determine NeuN^+^ cell density in each layer, and each NeuN^+^ cell was evaluated for cFos expression. Within the same tissue sections, we also evaluated the relative intensity of cFos expression in cortical layer II/III and V of demyelinated mice. Individual regions of interest were drawn around NeuN^+^ cFos^+^ cells within each layer. Background fluorescence was evaluated in five regions of interest drawn pseudorandomly in each layer, to encompass areas without cFos^+^ labeling. Mean gray value and integrated pixel density were measured using Image J (NIH). Corrected total cell fluorescence (CTCF) was calculated for cells within layer II/III or V of demyelinated mice using the following formula: [CTCF = Integrated Density − (Area of selected cell × Mean fluorescence of background readings)].

### Statistical analyses

Statistical comparisons were performed using Prism 9.5.1 (GraphPad). Data were evaluated using the Kolmogorov–Smirnov (KS) or Shapiro–Wilk (SW) normality tests. Normally distributed data were analyzed using an unpaired or paired *t* test or one- or two-way ANOVA followed by a Tukey's multiple-comparison test. A test for linear trend was performed following a one-way ANOVA where indicated. When unequal variance was observed between groups, determined by the Brown–Forsythe test or *F* test to compare variance, data were analyzed using a *t* test with a Welch's correction, or a Welch's ANOVA followed by a Dunnett's T3 multiple-comparisons post hoc test. Data that were not normally distributed were square root transformed to satisfy assumptions of normality and homoscedasticity of the variances followed by analysis by parametric tests where indicated. mEPSC data were analyzed in R (v4.4.2) using lme4, emmeans, and easystats packages for restriction maximum likelihood (REML) linear mixed models to determine the effect of cuprizone feeding with individual mouse included as a random effect term. *p* values < 0.05 were considered statistically significant. Statistical information is presented in the corresponding figure legend. The summary of the statistical analysis is shown in Tables 1 and 2. Data are presented as mean ± standard deviation unless otherwise specified.

**Table 1.** Statistical comparison of RABV-GFP^+^ neuron number and distribution per mouse in untreated versus cuprizone “control” cohorts

Comparison	Test	Statistics	Power (95% CI of difference)
RABV-GFP^+^ neurons (#)	Unpaired *t* test	*T* = 0.4342, *p* = 0.6772	−1,358 to 1,968
RABV-GFP^+^ cortical neurons (#)	Unpaired *t* test	*T* = 0.5216, *p* = 0.6181	−621.6 to 973.4
RABV-GFP^+^ cortical neurons (%)	Unpaired *t* test on sqrt transformed data	*T* = 0.003 *p* = 0.99	−2.122 to 2.116
RABV-GFP^+^ contralateral cortical neurons (#)	Unpaired *t* test	*T* = 0.2560, *p* = 0.8053	−128.9 to 160.2
RABV-GFP^+^ contralateral cortical neurons (%)	Unpaired *t* test	*T* = 0.5307, *p* = 0.6120	−6.843 to 4.334
RABV-GFP^+^ hippocampal neurons (#)	Unpaired *t* test	*T* = 0.2509, *p* = 0.8091	−890.0 to 1,101
RABV-GFP^+^ hippocampal neurons (%)	Unpaired *t* test on sqrt transformed data	*T* = 0.03 *p* = 0.97	−1.484 to 1.447
RABV-GFP^+^ contralateral hippocampal neurons (#)	Unpaired *t* test	*T* = 0.5837, *p* = 0.5777	−171.0 to 103.3
RABV-GFP^+^ contralateral hippocampal neurons (%)	Unpaired *t* test on sqrt transformed data	*T* = 1.74 *p* = 0.12	−2.551 to 0.3836
RABV-GFP^+^ contralateral neurons (#)	Unpaired *t* test	*T* = 0.1.338, *p* = 0.2228	−12.94 to 3.587
RABV-GFP^+^ thalamic/hypothalamic neurons (%)	Unpaired *t* test	*T* = 1.397, *p* = 0.2052	−0.7646 to 2.970
RABV-GFP^+^ neurons per cortical region (%)	Two-way ANOVA	interaction *F*_(5,42)_ = 0.4727, *p* = 0.7944; region *F*_(5,42)_ = 6.090, *p* = 0.0003; treatment *F*_(1,42)_ = 0.0180, *p* = 0.8941	Difference between treatments; −6.309 to 7.206
RABV-GFP^+^ neurons per hippocampal region (%)	Two-way ANOVA	interaction *F*_(3,28)_ = 1.835, *p* = 0.1638; region *F*_(3,28)_ = 2,962, *p* < 0.001; treatment *F*_(1,28)_ = 3,585e-009, *p* < 0.9999	Difference between treatments; −1.711 to 1.711
RABV-GFP^+^ cortical layer distribution (%)	Two-way ANOVA	Interaction *F*_(3,28)_ = 0.353, *p* = 0.7836, cortical layer *F*_(3,28)_ = 69.46, *p* < 0.0001, treatment *F*_(1,28) _= 1.164 × e-005, *p* = 0.9973	Difference between treatments; −4.944 to 4.961

**Table 2.** Summary of main statistics

	Figure	Graph	Data structure	Type of test	*p* value	Power (95% CI of difference)
a	1	K	Sqrt transformed	One-way ANOVA:	<0.0001	
				Tukey's post hoc test	CTX versus CC <0.0001	
					CTX versus HIP 0.0528	
					CC versus HIP <0.0001	
b		L	Sqrt transformed	One-way ANOVA;	0.012	
				Tukey's post hoc test	1 DPI versus 7DPI 0.01	
					2DPI versus 7 DPI 0.058	
				Post hoc test for linear trend	0.0004	
c	2	B	Sqrt transformed	Welch's *t* test	0.012	10.21 to 43.62
d		I	Normally distributed	Welch's ANOVA	0.0045	
				Dunnett's post hoc test	HIP versus TH/HYTH; 0.0136	23.91 to 104.2
e		J	Normally distributed	Two-way ANOVA	Hemisphere <0.0001	−78.02 to −68.59
				Tukey's post hoc tests	Ipsi versus contra all regions; 0.0001	−82.86 to −52.60
					Ipsi versus CTX; <0.0001	−70.41 to −40.16
					Ipsi versus contra HIP; <0.0001	−85.34 to −55.09
f		K	Normally distributed	One-way ANOVA	<0.0001	
				Tukey's post hoc tests	CA1 versus CA2; <0.0001	78.62 to 92.17
					CA1 versus CA3; <0.0001	82.92 to 96.47
					CA1 versus subiculum; <0.0001	84.56 to 98.11
g		L	Normally distributed	One-way ANOVA	0.0012	
				Tukey's post hoc tests	Somatosensory versus auditory; 0.0066	6.726 to 60.08
					Somatosensory versus cingulate; 0.0014	11.46 to 64.82
					Somatosensory versus insular; 0.0015	11.40 to 64.76
					Somatosensory versus rhinal; 0.0016	11.06 to 64.42
h		P	Normally distributed	One-way ANOVA	<0.0001	
				Tukey's post hoc test	II/III versus V; 0.0008	−47.23 to −13.64
					IV versus V; 0.0003	−50.38 to −16.78
					V versus VI; <0.0001	26.68 to 60.28
I	2-1	D	Normally distributed	Paired *t* test	<0.0001	98.93 to 100.1
J	3	E	Normally distributed	One-way ANOVA	<0.0001	
				Tukey's post hoc tests	Control versus demyelinated; <0.0001	19.59 to 32.73
					Demyelinated versus remyelinating; <0.0001	−33.92 to −20.79
k		J	Normally distributed	Two-way ANOVA	Region <0.0001	−0.1558 to 0.2438
				Tukey's post hoc tests	Control: CTX versus HIP; 0.0036	0.1906 to 1.028
					Demyelinated: CTX versus CC; 0.0053	−1.002 to −0.1650
					Demyelinated: CC versus HIP; 0.0001	0.4077 to 1.245
l		K	Normally distributed/nonparametric	Kolmogorov–Smirnov test	0.0021	
m		L	Normally distributed	Welch's *t* test; *F* test to compare variance	0.0203	
n		M	Normally distributed/nonparametric	Kolmogorov–Smirnov test	0.0002	
o	4	A	Sqrt transformed	One-way ANOVA	0.0019	
				Tukey's post hoc test	Control versus demyelinating: 0.0309	
				Tukey's post hoc test	Control versus remyelinating; 0.0023	
p	5	F	Sqrt transformed	One-way ANOVA	0.0163	
				Tukey's post hoc test	Control versus demyelinated; 0.0232	
q		G	Sqrt transformed	One-way ANOVA	0.0003	
				Tukey's post hoc tests	Control versus demyelinated; 0.0167	1.051 to 10.97
					Control versus remyelinating; 0.0003	3.895 to 12.21
r		H	Sqrt Transformed	One-way ANOVA	0.0116	
				Tukey's post hoc tests	Control versus demyelinated; 0.0336	0.2532 to 6.662
					Control versus remyelinating; 0.0270	0.3414 to 5.962
s		I	Sqrt transformed	One-way ANOVA	0.0026	
				Tukey's post hoc test	Control versus remyelinating; 0.0022	2.558 to 11.17
t		J	Sqrt transformed	One-way ANOVA	0.0050	
				Tukey's post hoc test	Control versus demyelinating; 0.0744	−0.1149 to 2.709
					Control versus remyelinating 0.0052	0.5368 to 3.014
u		K	Sqrt transformed	One-way ANOVA	0.0003	
				Tukey's post hoc test	Control versus demyelinating; 0.0167	1.051 to 10.97
					Control versus remyelinating 0.0003	3.895 to 12.21
v		M	Normally distributed	Two-way ANOVA	Region; 0.0108	
				Tukey's post hoc tests	Retrosplenial control versus remyelinating; <0.0001	−43.38 to −13.03
					Somatosensory control versus remyelinating; 0.0333	1.037 to 31.39
w	6	D	Normally distributed	Two-way ANOVA	Cortical layer 0.009; treatment 0.0006	
				Tukey's post hoc tests	Control versus demyelinated layer V; 0.0129	18.61 to 187.4
					Control versus remyelinating layer V; >0.0001	66.82 to 214.9
					Control layer II/III versus V; 0.0015	−179.3 to −33.77
					Control layer IV versus V; 0.0005	−187.6 to −42.00
					Control layer V versus VI; 0.0005	67.22 to 212.8
x		E	Normally distributed	Two-way ANOVA	Cortical layer <0.0001; interaction 0.0062	
				Tukey's post hoc tests	Control versus demyelinated layer V; 0.0219	1.716 to 26.59
					Control versus remyelinating layer V; 0.0376	−27.38 to −0.6611
					Control layer II/III versus V; <0.0001	−42.10 to −20.65
					Control layer II/III versus VI; 0.0203	1.420 to 22.87
					Control layer IV versus V; <0.0001	−45.22 to −23.77
					Control layer V versus VI; <0.0001	32.79 to 54.24
					Demyelinated layer II/III versus IV; 0.013	3.105 to 35.28
					Demyelinated layer II/III versus VI; 0.0119	3.305 to 35.48
					Demyelinated layer IV versus V; 0.0012	−40.08 to −7.900
					Demyelinated layer V versus VI; 0.0011	8.100 to 40.28
					Remyelinating layer II/III versus V; <0.0001	−42.74 to −16.46
					Remyelinating layer IV versus V; <0.0001	−55.79 to −29.52
					Remyelinating layer V versus VI; <0.0001	23.72 to 49.99
aa	6-1	A	Normally distributed	Two-way ANOVA	Treatment 0.0006 Cortical layer <0.0001 Interaction 0.046	
				Tukey's posttest	Layer II/III: control versus demyelinated 0.0025	287.9 to 1,080
					Control: Layer II/III versus V < 0.0001	761.1 to 1,553
					Demyelinated: Layer II/III versus 5 < 0.0014	350.5 to 1,143
		B	Normally distributed	Two-way ANOVA	Treatment 0.0004	
				Tukey's posttest	Layer II/III: control versus demyelinated 0.008	−1,203 to −227.2
					Layer V: control versus demyelinated 0.023	−1,168 to −92.64
		C	Normally distributed	Two-way ANOVA	Treatment 0.0002	
				Tukey's posttest	Layer II/III: control versus demyelinated 0.012	−48.84 to −6.23
					Layer V: control versus demyelinated 0.006	−51.61 to −9.007
		D	Normally distributed	Paired *t* test	0.0629	−1,675 to 104.7

Data were included/excluded from analysis based on a set of predetermined criteria. Specifically, injection of the SADΔG-GFP-(EnvA) virus into the subcortical white matter of *RphiGT* control mice resulted in the nonspecific labeling of 15 ± 7 neurons, and previous studies have estimated the minimum number of synaptic inputs received by individual callosal OPCs to be between ∼11 and 24 (Kukley et al., 2007; Mount et al., 2019). Therefore, if the number of RABV-GFP^+^ neurons labeled in *Pdgfrα-CreER^T2^ :: RphiGT* experimental mice was <2-fold greater than 15 ± 7, it was assumed that insufficient viral labeling of OPCs or transsynaptic labeling of connected neurons occurred in that mouse. Based on this assumption, *n* = 1 *Pdgfrα-CreER^T2^ :: RphiGT* transgenic mouse from the cuprizone demyelinated cohort, and *n* = 2 *Pdgfrα-CreER^T2^ :: RphiGT* transgenic mice from the remyelination cohort were excluded and not used to analyze the regional distribution of RABV-GFP^+^ neurons. Additionally, *n* = 1 *Pdgfrα-CreER^T2^ :: RphiGT* mouse from the remyelination cohort was not used to analyze the regional distribution of RABV-GFP^+^ hippocampal neurons, as no RABV-GFP^+^ neurons were identified within the hippocampus of that mouse.

## Results

### SADΔG-GFP-(EnvA) infected OPCs decline in number within 7 d

To characterize the population of neurons that synapse onto OPCs in the healthy adult mouse corpus callosum, we labeled OPCs with the glycoprotein-deleted SADΔG-GFP-(EnvA) rabies virus. This virus has been modified to restrict infection to cells that express TVA, the cognate receptor for EnvA. Furthermore, deletion of the gene encoding the rabies virus glycoprotein (RVG) has rendered the virus unable to cross the synapse, unless RVG is expressed by the infected cell. Delivery of tamoxifen to *Pdgfrα-CreER^T2^ :: RphiGT* transgenic mice induces the expression of TVA and RVG in PDGFRα^+^ OPCs, renders OPCs susceptible to infection and labeling by the SADΔG-GFP-(EnvA) virus, and allows the monosynaptic transfer of the virus to label neurons that directly synapse with the labeled OPCs in vivo (Mount et al., 2019).

Previous studies have demonstrated that the in vivo injection of SADΔG-GFP-(EnvA) virus can label some neurons and glia around the injection site, even in the absence of Cre-induced TVA expression (Marshel et al., 2010; Rancz et al., 2011; Takatoh et al., 2013). Therefore, we first delivered tamoxifen to P42 C57BL/6 (*n* = 3) and *RphiGT* control mice (*n* = 4) and injected SADΔG-GFP-(EnvA) into the corpus callosum at P49. After 7 d, RABV-GFP^+^ cells were not detected in C57BL/6 injected brains. The small number of RABV-GFP^+^ cells (46 ± 14) detected in the brains of *RphiGT* control mice were proximal to the injection site, but spanned the corpus callosum, hippocampus, and cortex (Extended Data Fig. 1-1*A*). A subset of these cells were identified as RABV-GFP^+^ NeuN^+^ neurons (15 ± 7 cells) and had the distinct morphology of excitatory cortical pyramidal neurons (Extended Data Fig. 1-1*B–D*), and fewer still (∼1–2 per brain) were interneurons. The remaining RABV-GFP^+^ cells were glia, including OLs (18 ± 8 cells; Extended Data Fig. 1-1*E–G*) and astrocytes (7 ± 6 cells; Extended Data Fig. 1-1*H–J*). Of note, RABV-GFP^+^ cells did not express the OPC marker PDGFRα, indicating that OPCs lacking the TVA receptor are not prone to nonspecific labeling by SADΔG-GFP-(EnvA) (Extended Data Fig. 1-1*K*).

**Figure 1.**
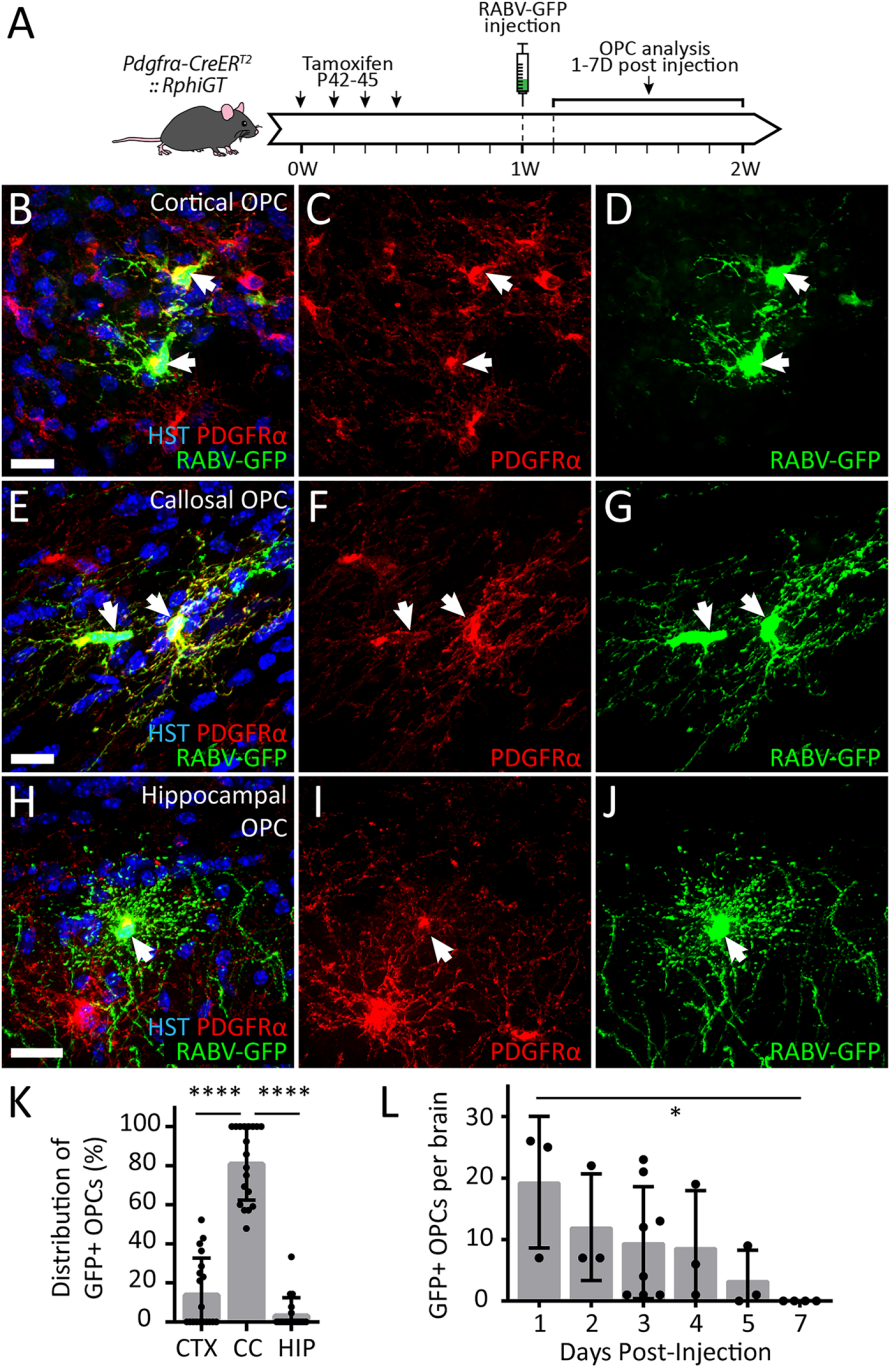
RABV-GFP^+^ OPCs are located in the corpus callosum, cortex, and hippocampus of *Pdgfrα-CreER^T2^ :: RphiGT* transgenic mice. ***A***, Experimental time-course schematic. *Pdgfrα-CreER^T2^ :: RphiGT* transgenic mice received tamoxifen from P42–45 and were injected with the SADΔG-GFP-(EnvA) virus at P49. Labeled RABV-GFP^+^ OPCs were visualized 1, 2, 3, 4, 5, and 7 d postinjection. ***B–J***, Representative compressed confocal images of RABV-GFP^+^ (green) PDGFRα^+^ (red) OPCs, with Hoechst 33342 labeled nuclei (HST; blue), in the cortex (CTX; ***A***–***C***), corpus callosum (CC; ***D–F***), and hippocampus (HIP; ***G–I***) of adult *Pdgfrα-CreER^T2^ :: RphiGT* transgenic mice injected with the SADΔG-GFP-(EnvA) virus. Scale bars represent 20 µm. ***K***, The proportion of RABV-GFP^+^ OPCs within the CTX, CC, and HIP of individual *Pdgfrα-CreER^T2^ :: RphiGT* transgenic mice (*n* = 19). One-way ANOVA *F*_(2,54)_ = 79.93, *p* < 0.0001; data were square root transformed for analysis to satisfy the assumptions of normality and homoscedasticity of the variances. ***K***, Quantification of the total number of RABV-GFP^+^ OPCs in *Pdgfrα-CreER^T2^ :: RphiGT* nice (*n* = 24) at 1, 2, 3, 4, 5, and 7 days postinjection. One-way ANOVA *F*_(5,18)_ = 4.051, *p* = 0.012, linear trend: *F*_(5,18)_ = 19.20, *p* = 0.0004, *R*^2^ = 0.94. Data were square root transformed for analysis to satisfy the assumptions of normality and homoscedasticity of the variances. Error bars display mean ± SD. *p ≤ 0.05, ****p ≤ 0.0001 by Tukey's multiple-comparison posttest. Extended data Figure 1-1 supports Figure 1.

In contrast, when SADΔG-GFP-(EnvA) was injected into the corpus callosum of P49 *Pdgfrα-CreER^T2^ :: RphiGT* transgenic mice (Fig. 1*A*), PDGFRα^+^ OPCs within the cortex (Fig. 1*B–D*), corpus callosum (Fig. 1*E–G*), and hippocampus (Fig. 1*H–J*) became labeled with RABV-GFP. In total, 207 RABV-GFP^+^ OPCs were identified across *n* = 24 *Pdgfrα-CreER^T2^ :: RphiGT* mice analyzed 1–7 d postinjection. Approximately 82% of these were located within the corpus callosum; ∼15% within the cortex, typically along the injection tract; and ∼4% within the hippocampal CA1 or alveus, in close proximity to the corpus callosum (Fig. 1*K*).

10.1523/ENEURO.0113-25.2025.f1-1 Figure 1-1 **Non-specific uptake of the SADΔG-GFP-(EnvA) virus *in vivo*** (A) Compressed confocal image of a coronal brain section from a P42+14 *RphiGT* control mouse 7-days post-injection of the SADΔG-GFP-(EnvA) virus (green) into the corpus callosum (CC), with Hoechst 33342 nuclear stain (HST, blue). The microinjection tract (red dashed line), cortex (CTX), CC, cingulum (Cg), and hippocampus (HIP) are indicated on the image. Solid arrows highlight RABV-GFP^+^ cortical neurons. Hollow arrows highlight RABV-GFP^+^ OLs. (B-D) Compressed confocal image showing a RABV-GFP^+^ (green) NEUN^+^ (red) cortical pyramidal neuron, with a HST^+^ nucleus (blue). (E-G) Compressed confocal image showing a RABV-GFP^+^ (green) ASPA^+^ (red) callosal OL, with a HST^+^ nucleus (blue). (H-J) Compressed confocal image showing a RABV-GFP^+^ (green) GFAP^+^ (red) cortical astrocyte, with a HST^+^ nucleus (blue). (K) Quantification of the total number of RABV-GFP^+^ OPCs, neurons, OLs, astrocytes, and unidentified cells per P42+14 *RphiGT* (n = 4) control mouse (mean ± SD). Scale bars represent 200 µm (A) or 20 µm (B-H). Download Figure 1-1, TIF file.

The number of RABV-GFP^+^ PDGFRα^+^ OPCs declined over time: 19 ± 11 RABV-GFP^+^ OPCs were detected per mouse at 1 d; 12 ± 9 at 2 d; 10 ± 9 at 3 d; 9 ± 9 at 4 d; and 4 ± 5 at 5 d and by 7 d postinjection, RABV-GFP^+^ OPCs could no longer be detected (Fig. 1*L*). While the SADΔG-GFP virus has been reported to induce neuronal cell death after ∼16 d (Wickersham et al., 2007), the loss of RABV-GFP^+^ OPC within 7 d of infection suggests that OPCs are less tolerant of viral amplification than neurons. The loss of RABV-GFP^+^ OPCs by 7 d postinfection prevents the direct association of single RABV-GFP^+^ OPCs with specific neuronal subset at 7 d but does not preclude a population level characterization of the synaptically connected neurons.

### Callosal OPCs preferentially synapse with from ipsilateral pyramidal neurons projecting from the CA1 region of the hippocampus and layer V of the cortex

**Figure 2.**
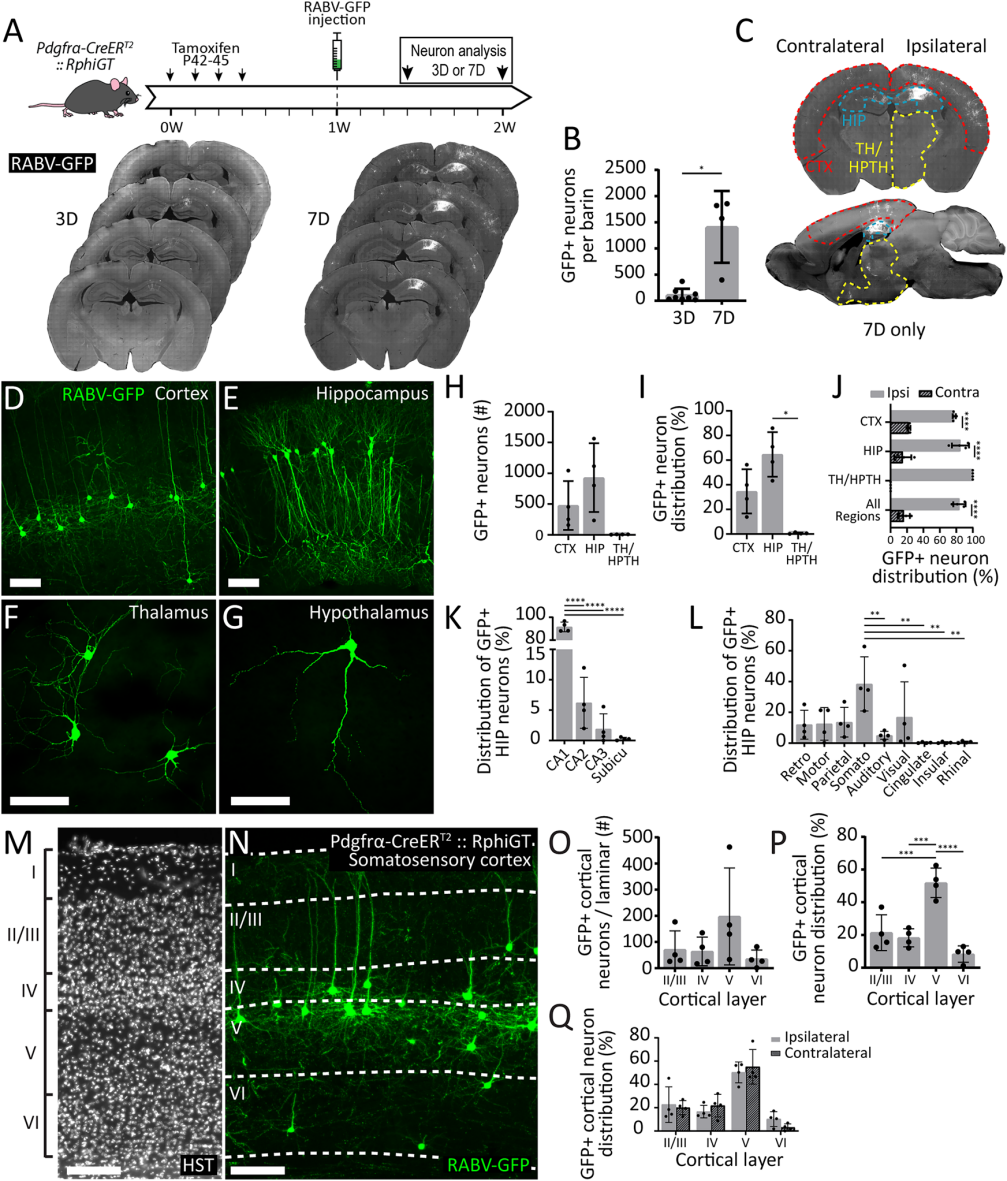
Callosal OPCs primarily receive synaptic input from ipsilateral pyramidal neurons in cortical layer V. ***A***, Experimental time-course schematic and representative compressed confocal image of serial coronal brain sections from *Pdgfrα-CreER^T2^ :: RphiGT* transgenic mice 3 and 7 d postinjection (***D***) of the SADΔG-GFP-(EnvA) virus into the corpus callosum. Images encompass the injection site and display RABV-GFP (white). ***B***, Quantification of the total number of RABV-GFP^+^ neurons 3 and 7 d after the SADΔG-GFP-(EnvA) virus was delivered into the corpus callosum of P42 + 7 *Pdgfrα-CreER^T2^ :: RphiGT* mice (*n* = 8 at 3 DPI, 4 at 7 DPI; mean ± SD). Welch's *t* test: *t* = 4652, df = 3.644, *p* = 0.012. Data were square root transformed for analysis to satisfy the assumptions of normality and homoscedasticity of the variances. ***C***, Compressed confocal image of RABV-GFP (white) labeling in a coronal and sagittal brain section from a P42 + 7 *Pdgfrα-CreER^T2^ :: RphiGT* transgenic mouse, 7 d postinjection of the SADΔG-GFP-(EnvA) virus into the corpus callosum. The cortex (CTX; red), hippocampus (HIP; blue), and thalamus plus hypothalamus (TH/HPTH; yellow) are outlined on the image. ***D–G***, Representative compressed confocal images of RABV-GFP^+^ (green) neurons in the cortex (***D***), hippocampus (***E***), thalamus (***F***), and hypothalamus (***G***) 7 d after injecting the SADΔG-GFP-(EnvA) virus into the corpus callous of P42 + 7 *Pdgfrα-CreER^T2^:: RphiGT* transgenic mice. ***H***, Quantification of the total number of RABV-GFP^+^ neurons in the CTX, HIP, and TH/HPTH of each *Pdgfrα-CreER^T2^ :: RphiGT* transgenic mouse (*n* = 4). Welch's ANOVA: *F*_(2,4)_ = 7.096, *p* = 0.0483. ***I***, The proportion of RABV-GFP^+^ neurons in the CTX, HIP, and TH/HPTH of each *Pdgfrα-CreER^T2^ :: RphiGT* transgenic mouse (*n* = 4). Welch's ANOVA: *F*_(2,4.009)_ = 27.550, *p* = 0.005. ***J***, The proportion of ipsilateral and contralateral RABV-GFP^+^ neurons within the whole brain, CTX, HIP, and TH/HPTH of *Pdgfrα-CreER^T2^ :: RphiGT* transgenic mice (*n* = 4). Two-way ANOVA: interaction *F*_(2,18)_ = 2.301, *p* = 0.129; region *F*_(2,18)_ = 3.670 × 10^−10^, *p* > 0.999; hemisphere *F*_(1,18) _= 447.4, *p* < 0.0001. ***K***, The proportion of hippocampal RABV-GFP^+^ neurons identified in hippocampal subregions of individual *Pdgfrα-CreER^T2^ :: RphiGT* transgenic mice (*n* = 4). One-way ANOVA: *F*_(3,12)_ = 759.3, *p* < 0.0001. ***L***, The proportion of cortical RABV-GFP^+^ neurons identified in each cortical region of individual *Pdgfrα-CreER^T2^:: RphiGT* transgenic mice (*n* = 4). One-way ANOVA: *F*_(8,27)_ = 4.642, *p* = 0.001. ***M***, Compressed confocal image of the cortex in which Hoechst 33342 nuclear stain (HST; white) was used to identify each cortical layer (I, II/III, IV, V, and VI). ***N***, Compressed confocal image of the somatosensory cortex of a P42 + 14 *Pdgfrα-CreER^T2^ :: RphiGT* transgenic mouse 7 d postinjection of the SADΔG-GFP-(EnvA) virus into the corpus callosum, showing RABV-GFP^+^ (green) cortical neurons distributed across the cortical laminar. ***O***, Quantification of the total number of RABV-GFP^+^ cortical neurons in cortical layers II/III, IV, V, and VI of individual *Pdgfrα-CreER^T2^ :: RphiGT* transgenic mice (*n* = 4). One-way ANOVA: *F*_(3,12)_ = 1.904, *p* = 0.183. ***P***, The proportion of RABV-GFP^+^ cortical neurons in cortical layers II/III, IV, V, and VI of individual *Pdgfrα-CreER^T2^ :: RphiGT* transgenic mice (*n* = 4). One-way ANOVA: *F*_(3,12)_ = 21.99, *p* < 0.0001. ***Q***, The proportion of ipsilateral and contralateral RABV-GFP^+^ cortical neurons within cortical layers II/III, IV, V, and VI, in individual *Pdgfrα-CreER^T2^ :: RphiGT* transgenic mice (*n* = 4). Two-way ANOVA: interaction *F*_(3,24)_ = 0.772, *p* = 0.521; laminar *F*_(3,24)_ = 33.13, *p* < 0.0001; and hemisphere *F*_(1,24)_ = 2.839 × 10^−5^, *p* = 0.996. Data are expressed as mean ± SD. Closed circles represent individual animals. Scale bars represent 100 µm (***D–G*** and ***M***, ***N***). *p ≤ 0.05, **p ≤ 0.01, ***p ≤ 0.001, ****p ≤ 0.0001 by Dunnett's T3 (***I***) or Tukey's (***J–L*** and ***P***) multiple-comparison posttest. Extended Data Figure 2-1 supports Figure 2.

In contrast to RABV-GFP^+^ OPCs, RABV-GFP^+^ neurons were not detected in *Pdgfrα-CreER^T2^ :: RphiGT* mice until 3 d postinjection and increased in number ∼13-fold between 3 (111 ± 119 cells) and 7 days (1,413 ± 687 cells; Fig. 2*A*,*B*). This was significantly higher than the number of RABV-GFP^+^ neurons in *RphiGT* mice (compare Extended Data Fig. 1-1*K* and Fig. 2*B*; Welch's *t* test: *t* = 4.070, *p* = 0.027), consistent with the successful transsynaptic trafficking of SADΔG-GFP from infected OPCs to presynaptic neurons in *Pdgfrα-CreER^T2^ :: RphiGT* mice. Based on the location of RABV-GFP^+^ neuronal soma at 7 d, we deduced that the infected population of callosal and CA1/alveus OPCs primarily receive synaptic input from cortical (478 ± 397 RABV-GFP^+^ neurons per mouse, mean ± SD) or hippocampal neurons (929 ± 558 RABV-GFP^+^ neurons per mouse, mean ± SD; Fig. 2*C–H*), respectively. A total of 35 ± 18% of the RABV-GFP^+^ neurons identified in each brain were in the cortex, 65 ± 18% in the hippocampus, and <1% in the thalamic and hypothalamic nuclei (Fig. 2*I*). Then, 84 ± 7% of all labeled neurons were ipsilateral to the injection site, including ∼78% of the RABV-GFP^+^ cortical and ∼85% of the hippocampal neurons (Fig. 2*C*,*J*). Thalamic and hypothalamic connectivity was exclusively ipsilateral (Fig. 2*J*).

10.1523/ENEURO.0113-25.2025.f2-1 Figure 2-1 **Callosal OPCs receive minimal synaptic input from parvalbumin interneurons** (A-C) Representative compressed confocal image of a RABV-GFP^+^ (green), parvalbumin^+^ (PV; red) interneuron, with a Hoechst 33342 (HST) labelled nucleus (blue) in the cortex of a P42+14 *Pdgfrα-CreER^T2^ :: RphiGT* transgenic mouse 7-days after SADΔG-GFP-(EnvA) virus was injected into the corpus callosum. Scale bar represents 20 μm. (D) The proportion of RABV-GFP+ neurons that co-label for PV in *Pdgfrα-CreER^T2^ :: RphiGT* transgenic mice (n = 4; mean ± SD). Paired t-test; t (3) = 537.4, p<0.0001. ****p ≤ 0.0001. Download Figure 2-1, TIF file.

To examine the regional distribution of RABV-GFP^+^ neurons within the cortex and hippocampus, data from both hemispheres were combined. Within the hippocampus, 92 ± 4% of RABV-GFP^+^ neurons were found to reside in the CA1 region, with a smaller proportion in CA2 (6 ± 4%) or CA3 (2 ± 3%; Fig. 2*K*). Of the cortical neurons that synapsed onto callosal OPCs, 39 ± 18% were located in the somatosensory cortex; 17 ± 23% in the visual cortex, and 14 ± 10% in the parietal cortex, with RABV-GFP^+^ neurons also being identified in the retrosplenial (12 ± 9%), motor (13 ± 11%), and auditory cortices (39 ± 18%), but rarely (<1% per region) within the cingulate, insular, or rhinal cortices (Fig. 2*L*).A small proportion of RABV-GFP^+^ neurons were parvalbumin^+^ interneurons (0.24 ± 0.19%, ∼ 1–4 cells per brain; Extended Data Fig. 1-2*A–D*), but the majority (>99%) had the morphology of glutamatergic projection neurons.

Most axons that traverse the mouse corpus callosum project from neurons in cortical layers II/III and V, and a small minority project from neurons in layers IV and VI (Wise, 1975; Fame et al., 2011; Chovsepian et al., 2017). A laminar distribution analysis was possible for RABV-GFP^+^ cortical neurons spanning the parietal, somatosensory, auditory, and visual cortices (74 ± 15% of all RABV-GFP^+^ cortical neurons per mouse), as their laminar organization allowed identification of layers I, II/III, IV, V, and VI, using Hoechst nuclear staining (Fig. 2*M*). RABV-GFP^+^ cortical neurons were distributed across layers II/III, IV, V, and VI in all mice (Fig. 2*N*,*O*). Unsurprisingly, no RABV-GFP^+^ neurons were located in layer I, a region predominantly composed of interneurons (Larkum, 2013) that do not extend their axons into the corpus callosum (Chovsepian et al., 2017). The majority of RABV-GFP^+^ cortical neurons were located within layer V and were morphologically classified as pyramidal neurons (52 ± 9%; 198 ± 185 neurons per brain). The remaining RABV-GFP^+^ cortical neurons were equally distributed across layers II/III, IV, and VI (Fig. 2*N–P*). While fewer RABV-GFP^+^ neurons were in the contralateral cortex, their distribution across cortical layers was the same (Fig. 2*Q*). As neurons originating in layer II/III comprise the largest population of transcallosal axons in the mouse brain (Fame et al., 2011; Chovsepian et al., 2017), these data suggest that in the adult mouse corpus callosum, OPCs preferentially form synaptic connections with layer V pyramidal neurons.

### Cuprizone-mediated demyelination reduces the number of neurons that synapse with OPCs

**Figure 3.**
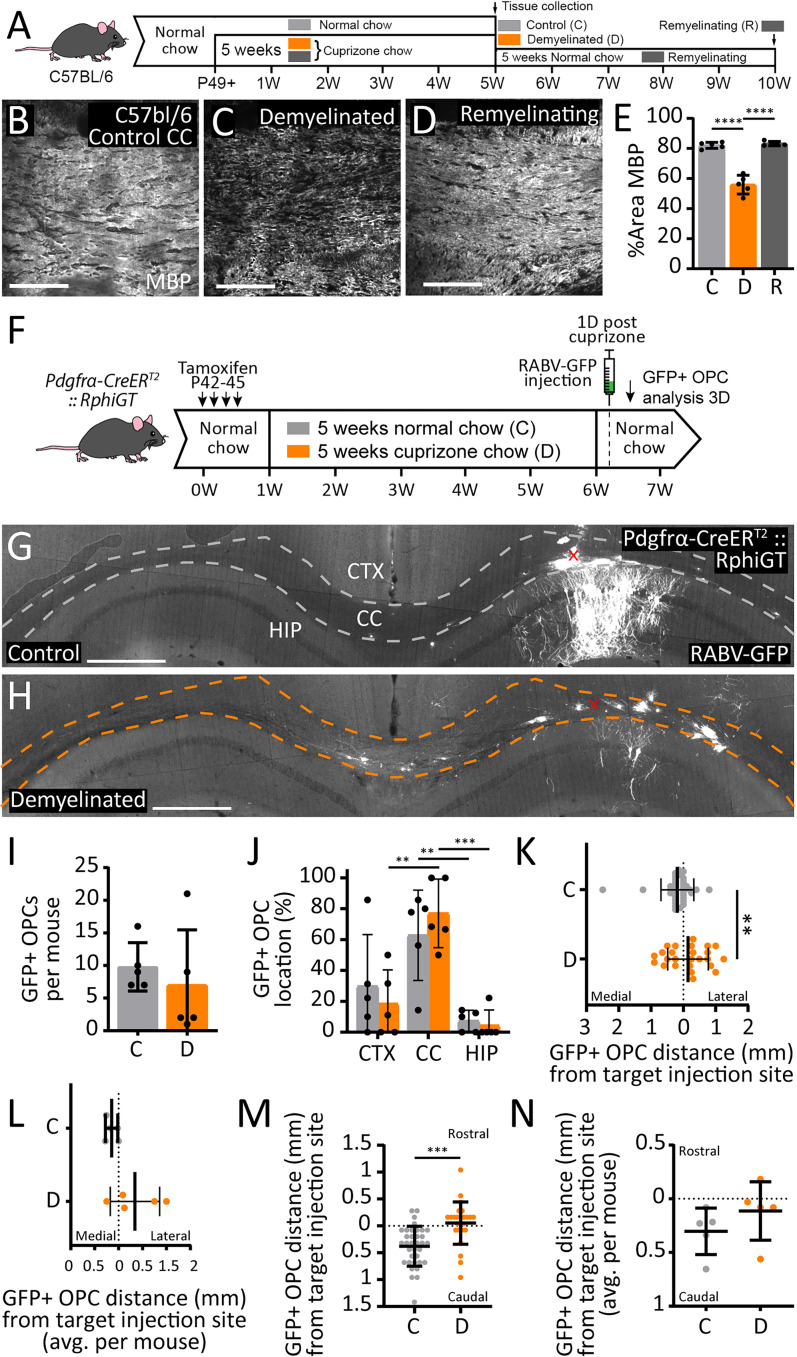
Cuprizone induces reversible demyelination of the corpus callosum and alters the distribution but not the number of RABV-GFP^+^ OPCs. ***A***, Experimental time-course schematic. C57BL/6 control mice were retained on normal chow and tissue collected at P84. Demyelinated mice received 0.2% (w/w) cuprizone chow from P49 and tissue was collected at P84. Remyelinating mice received 0.2% (w/w) cuprizone chow P49–P84 and were returned to normal chow until tissue was collected at P119. ***B–D***, Confocal images of the medial corpus callosum (CC) in coronal brain sections immunolabeled to detect MBP (white), taken from a control (***A***), demyelinated (***B***), and remyelinating (***C***) adult C57bL/6 mice. ***E***, Quantification of the area of the medial CC labeled for MBP in individual control, demyelinated, and remyelinating C57bL/6 mice (*n* = 5 per group; mean ± SD). One-way ANOVA: *F*_(2,12)_ = 78.92, *p* < 0.0001. ***F***, Experimental time-course schematic. *Pdgfrα-CreER^T2^ :: RphiGT* mice received tamoxifen from P42–45. Control mice were retained on control diet for the duration of the experiment. Demyelinated mice were transferred to 0.2% (w/w) cuprizone chow from P49 to P84. All mice were on a normal diet and received the SADΔG-GFP-(EnvA) virus at P85. RABV-GFP^+^ OPCs were analyzed at P87 (3D). ***G–H***, Compressed confocal image of coronal brain section from a control (***A***) or cuprizone demyelinated (***B***) adult *Pdgfrα-CreER^T2^ :: RphiGT* transgenic mice 3D after the SADΔG-GFP-(EnvA) virus (white) was injected into the corpus callosum (CC). The cortex (CTX), CC, hippocampus (HIP), and the location of viral microinjection (marked “X”) are indicated on the images. ***I***, Quantification of the total number of RABV-GFP^+^ PDGFRα^+^ OPCs per control or demyelinated *Pdgfrα-CreER^T2^ :: RphiGT* SADΔG-GFP-(EnvA) injected transgenic mouse (*n* = 5 per group). Unpaired *t* test: *t* = 0.678, *p* = 0.517. ***J***, The proportion of RABV-GFP^+^ OPCs located in the CTX, CC, and HIP in control or demyelinated *Pdgfrα-CreER^T2^ :: RphiGT* transgenic mice (*n* = 5 per group). Two-way ANOVA: region × treatment *F*_(2,24)_ = 0.715, *p* = 0.499; region *F*_(2,24)_ = 18.63, *p* < 0.0001; treatment *F*_(1,24)_ = 0.286, *p* = 0.597. Data were square root transformed for analysis to satisfy the assumptions of normality and homoscedasticity of the variances. ***K***, The distance (mm) of individual callosal and hippocampal RABV-GFP^+^ OPCs from the site of viral microinjection on the medial-lateral axis within control (*n* = 35 RABV-GFP^+^ OPCs) or demyelinated (*n* = 25 RABV-GFP^+^ OPCs) *Pdgfrα-CreER^T2^ :: RphiGT* transgenic mice (*n* = 5 mice per group). KS test, *p* = 0.0021. ***L***, The mean distance (mm) of each RABV-GFP^+^ OPC from the site of viral microinjection along the medial-lateral axis, per control or demyelinated *Pdgfrα-CreER^T2^ :: RphiGT* transgenic mouse (*n* = 5 per group). Welch's *t* test: *t* = 2.052, df = 4.503, *p* = 0.102. ***M***, The distance (mm) of individual callosal and hippocampal RABV-GFP^+^ OPCs from the site of viral microinjection on the rostral-caudal axis within control (*n* = 35 RABV-GFP^+^ OPCs) or demyelinated (*n* = 25 RABV-GFP^+^ OPCs) *Pdgfrα-CreER^T2^ :: RphiGT* transgenic mice cohorts (*n* = 5 mice per group). KS test, *p* = 0.0002. ***N***, The mean distance (mm) of each RABV-GFP^+^ OPC from the site of viral microinjection along the rostral-caudal axis, in control or demyelinated *Pdgfrα-CreER^T2^ :: RphiGT* transgenic mouse (*n* = 5 per group). Welch's *t* test: *t* = 1.22, df = 7.617, *p* = 0.25. Data are expressed as mean ± SD. Closed circles represent individual animals (***E***, ***I–L***, ***N***), or individual OPCs (K,M). Scale bars represent 100 µm (***B–D***) or 500 µm (***G–H***). **p ≤ 0.01, ***p ≤ 0.001, ****p ≤ 0.0001 by Tukey's multiple-comparison posttest (***E***, ***J***).

To investigate the impact of demyelination on neuron→OPC synaptic connectivity, P49 *Pdgfrα-CreER^T2^ :: RphiGT* transgenic mice were maintained on a control diet or transferred to 0.2% (w/w) cuprizone for 5 weeks to induce demyelination (Nguyen et al., 2024) prior to SADΔG-GFP-(EnvA) delivery. As cuprizone withdrawal allows significant spontaneous remyelination to occur over a 4 week period (Skripuletz et al., 2011; Gingele et al., 2020; Palavra et al., 2022), a separate cohort of demyelinated *Pdgfrα-CreER^T2^ :: RphiGT* mice were returned to a control diet, to support remyelination prior to SADΔG-GFP-(EnvA) virus delivery. In C57BL/6 mice, 5 weeks of cuprizone feeding induces a ∼31% decrease in MBP^+^ pixel coverage in the corpus callosum at ∼bregma −1.5 mm (Fig. 3*A–C*,*E*) and a 5 week remyelination period restores MBP^+^ pixel coverage in the corpus callosum to control levels (Fig. 3*D*,*E*).

When SADΔG-GFP-(EnvA) virus was injected into the corpus callosum of control or demyelinated P84 *Pdgfrα-CreER^T2^ :: RphiGT* transgenic mice (Fig. 3*F–H*), a similar number of PDGFRα^+^ OPCs became RABV-GFP^+^ in control (10 ± 4 cells) and demyelinated (7 ± 9 cells) mice (Fig. 3*I*), and most of the RABV-GFP^+^ OPCs were located within the corpus callosum (control 63 ± 26% vs demyelinated 77 ± 20%; Fig. 3*J*). Perhaps unsurprisingly, demyelination was associated with lateral extension of the OPC labeling along the corpus callosum (*F* test to compare variance *p* = 0.02; Fig. 3*K*,*L*) and a greater rostrocaudal distribution (Fig. 3*M*,*N*). This is likely the result of increased viral diffusion in the absence of myelin or the increased migration of infected OPCs.

**Figure 4.**
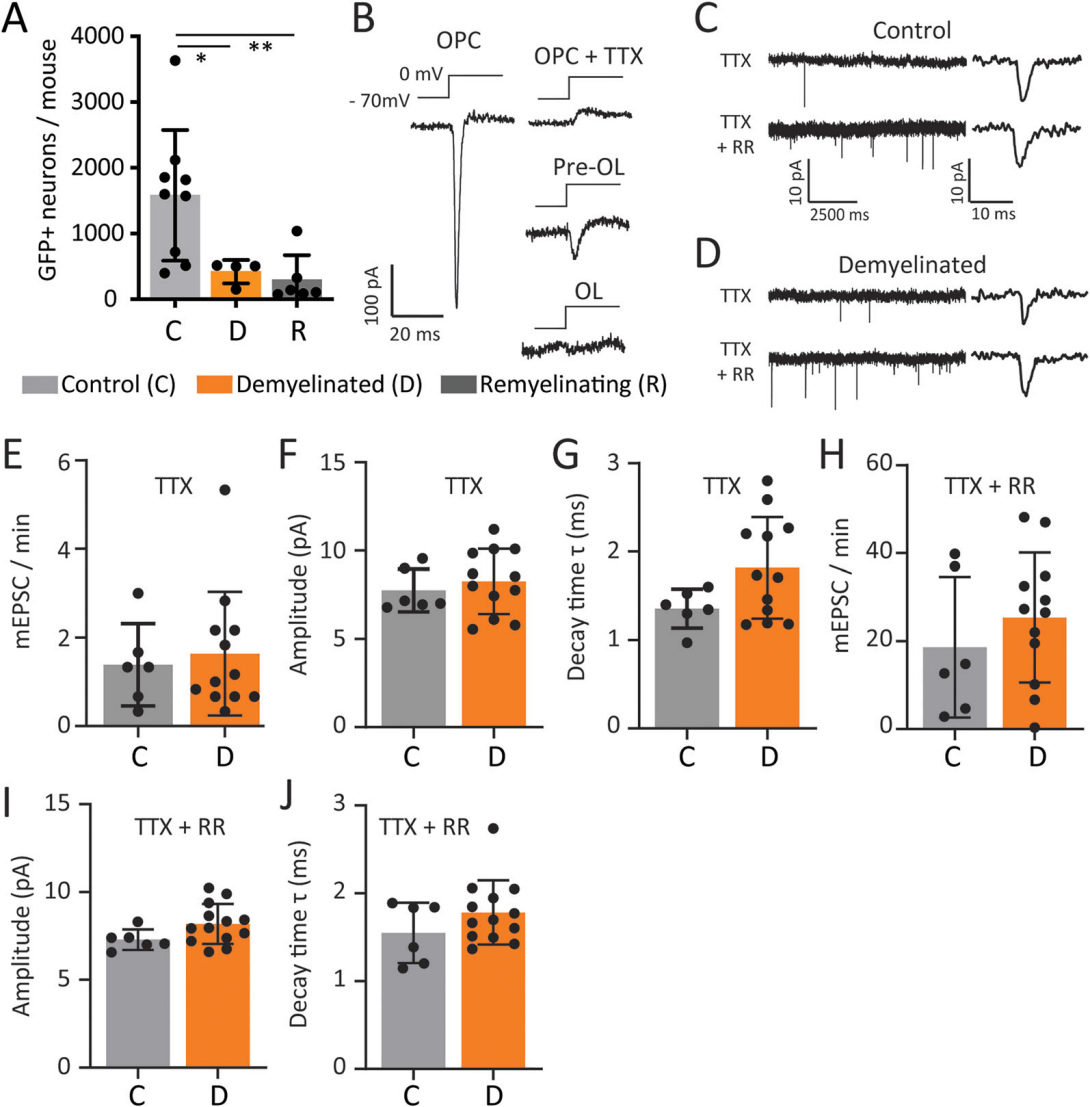
The number of RABV-GFP^+^ neurons detected in *Pdgfrα-CreER^T2^:: RphiGT* transgenic mice decreases following cuprizone demyelination. ***A***, Quantification of the total number of RABV-GFP^+^ neurons in *Pdgfrα-CreER^T2^ :: RphiGT* transgenic mice 7D after callosal injection with the SADΔG-GFP-(EnvA) virus in control, demyelinated or remyelinating mice (*n* ≥ 5 per group). One-way ANOVA: *F*_(2,16)_ = 9.486, *p* = 0.0019; with Tukey's posttest. Data were square root transformed for analysis to satisfy the assumptions of normality and homoscedasticity of the variances. ***B***, Representative traces of voltage-gated sodium currents evoked by a depolarizing step from −70 to 0 mV recorded from GFP^+^ OPCs (in the absence and presence of tetrodotoxin; TTX), newly born OLs (pre-OLs), and mature OLs, in the corpus callosum of acute brain slices generated from P85 *Pdgfrα-H2BGFP* transgenic mice. ***C***, ***D***, Example traces showing mEPSCs recorded from GFP^+^ callosal OPCs in acute brain slices generated from P85 *Pdgfrα-H2BGFP* transgenic mice following 5 weeks of control (***C***) or cuprizone (0.2% w/w) (***D***) diet, in the presence of TTX or TTX and the secretagogue ruthenium red (RR). Representative examples of individual ePSCs show the fast rise and decay times. ***E***, ***H***, The frequency of mEPSCs recorded from individual GFP^+^ callosal OPC of control or demyelinated mice. Recordings were made in the presence of TTX (***E***; REML, linear mixed effect ANOVA: *F*_(1,9.784)_ = 0.1211, *p* = 0.7352) or TTX and RR (***H***; REML linear mixed effect model: *F*_(1,16)_ = 0.795, *p* = 0.3857). ***F***, ***I***, The mean amplitude of mEPSCs recorded in individual callosal OPC of control or demyelinated mice. Recordings were made in the presence of TTX (F: REML, linear mixed effect ANOVA: *F*_(1,7.054)_ = 0.594, *p* = 0.465) or TTX and RR (***I***: REML, linear mixed effect ANOVA: *F*_(1,6.973)_ = 2.574, *p* = 0.152). ***G***, ***J***, The mean decay time for mEPSCs recorded in individual callosal OPC of control or demyelinated mice. Recordings were made in the presence of TTX (***G***: REML, linear mixed effect ANOVA: *F*_(1,8.602)_ = 3.299, *p* = 0.2042) or TTX and RR (REML, linear mixed effect ANOVA: *F*_(1,16)_ = 1.6083, *p* = 0.222). Data are expressed as mean ± SD. Closed circles represent individual animals (A = 9 control, 4 demyelinated, 6 remyelinating) or individual cells (***E–J***:6 control and 12 demyelinated OPCs). *p ≤ 0.05, **p ≤ 0.01.

To determine if the population of neurons that synapse with callosal OPCs was altered by demyelination, P85 control and demyelinated *Pdgfrα-CreER^T2^ :: RphiGT* mice were injected with SADΔG-GFP-(EnvA) and maintained on standard chow for 7 d to ensure robust neuronal RABV-GFP expression (P85 + 7D). We first compared the number and location of RABV-GFP^+^ neurons in P85 + 7D control mice (*n* = 5) with that of our previous P49 + 7D controls (*n* = 4; Fig. 2). While highly variable, we found that the number and distribution of RABV-GFP^+^ neurons was equivalent across cohorts (statistical comparison in Table 1), and data from the *n* = 9 control mice were pooled prior to comparison with the demyelinated mice. As the number of RABV-GFP^+^ neurons in control mice was highly variable (*F* test to compare variance, *p* = 0.0083), we performed a square root data transformation prior to data analysis. We determined that fewer RABV-GFP^+^ neurons were labeled in demyelinated mice compared with controls (Fig. 4*A*, Table 2). These data are consistent with OPCs forming fewer synapses with demyelinated axons.

The reduced number of RABV-GFP^+^ neurons in cuprizone-demyelinated mice is consistent with a previous report that white matter OPCs within demyelinated focal lesions receive fewer synaptic inputs (Etxeberria et al., 2010; Gautier et al., 2015; Sahel et al., 2015). To explore the possibility that OPCs receive fewer synaptic inputs in the demyelinated corpus callosum, we performed whole-cell patch-clamp electrophysiology of callosal OPCs in young adult *Pdgfrα-H2BGFP* mice that received a standard or cuprizone diet for 5 weeks. Mice were returned to normal chow for 1 day prior to whole-cell patch-clamp recordings from GFP^+^ OPCs in the body of the corpus callosum (∼bregma −1.5 mm) obtained in acute brain slices. The identity of GFP^+^ OPCs was confirmed by a membrane capacitance (Cm) <50 pF and the presence of a TTX-sensitive voltage-gated sodium currents >100 pA, evoked by a voltage step from −70 to 0 mV (Fig. 4*B*; Pitman et al., 2020). Cuprizone demyelination did not alter the membrane resistance or capacitance of OPCs (Table 3), and mEPSC frequency was also unchanged (recordings made in the presence of TTX; Fig. 4*C–E*). We would not expect a change in the composition of presynaptic neurons to impact mEPSC properties. This was confirmed by measurement of mEPSC amplitude and decay time (Fig. 4*F*,*G*). However, the low frequency of mEPSCs in OPCs restricted the number of events available for analysis. To overcome this limitation, OPCs were exposed to the secretagogue, ruthenium red, to increase the frequency of glutamate release in the presence of TTX. This increased mEPSC frequency significantly for control and demyelinated OPCs (Fig. 4*H*) and increased the number of mEPSCs that could be evaluated to confirm that mEPSC amplitude (Fig. 4*I*) and decay time (Fig. 4*J*) were unchanged by demyelination.

**Table 3.** Membrane properties of patched clamped callosal OPCs from control and demyelinated mice

Mean ± SD	Pipette resistance	Ra (Ω)	Rm (Ω)	Cm (pF)	*I*_Na_ (pA) −70 to 0 mV step
Control (*n* = 6 OPCs)	4.5 ± 0.9	4.7 ± 1.9	828 ± 600	26.3 ± 6.0	−229 ± 89
Demyelinated (*n* = 12 OPCs)	5.0 ± 0.9	8.9 ± 2.7	835 ± 707	25.3 ± 3.9	−231 ± 90

Mean ± SD.

### Callosal OPCs primarily receive synaptic input from ipsilateral cortical neurons in the demyelinated and remyelinating brain

**Figure 5.**
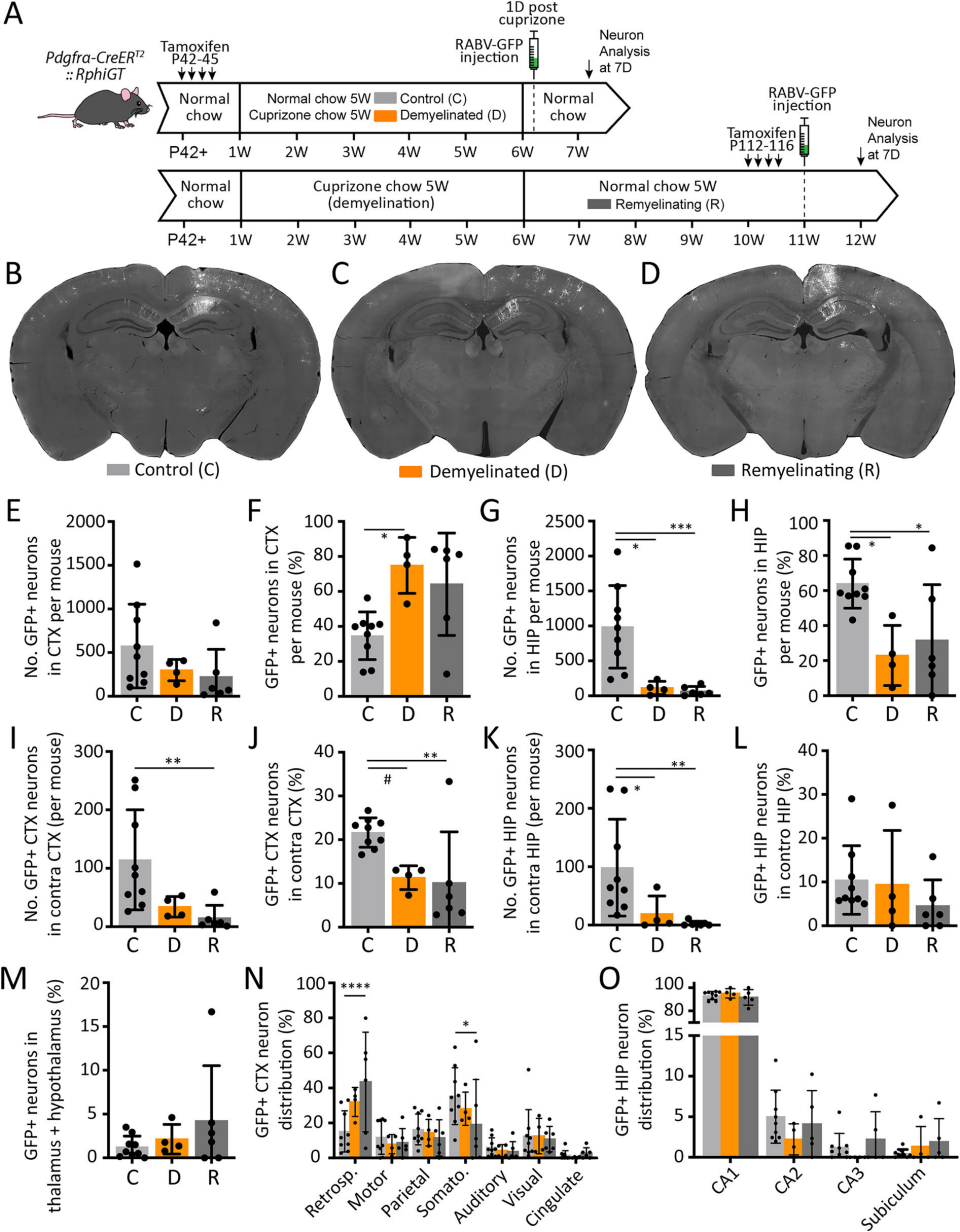
In the demyelinated brain callosal OPCs more frequently synapse with ipsilateral cortical neurons. ***A***, Experimental time-course schematic. *Pdgfrα-CreER^T2^ :: RphiGT* transgenic mice received tamoxifen from P42–45 and were split into three groups. Control mice received normal chow while demyelinated and remyelinating mice received 0.2% (w/w) cuprizone chow from P49 to P84. Control and demyelinated mice were injected with SADΔG-GFP-(EnvA) virus at P85 and analyzed at P92. Remyelinating mice were injected at P119 and analyzed at P126. ***B–D***, Representative coronal brain sections from control (***A***), demyelinated (***B***), and remyelinating (***C***) adult *Pdgfrα-CreER^T2^ :: RphiGT* transgenic mice 7 d postinjection of the SADΔG-GFP-(EnvA) virus (white) into the corpus callosum. ***E***, ***F***, Quantification of the total number of RABV-GFP^+^ neurons within the cortex of *Pdgfrα-CreER^T2^ :: RphiGT* control, demyelinated, and remyelinating mice (***E***; one-way ANOVA: *F*_(2,16)_ = 2.283, *p* = 0.134) and the proportion of RABV-GFP^+^ neurons that were found within the cortex (***F***; one-way ANOVA: *F*_(2,16)_ = 5.38, *p* = 0.0163; 9 controls, 4 demyelinated, and 6 remyelinating mice). Data were square root transformed for analysis to satisfy the assumptions of normality and homoscedasticity of the variances. ***G***, ***H***, Quantification of the total number of RABV-GFP^+^ neurons in the hippocampus of *Pdgfrα-CreER^T2^ :: RphiGT* control, demyelinated, and remyelinating mice (***G***; one-way ANOVA: *F*_(2,16)_ = 13.30, *p* = 0.0003) and the proportion of neurons located within the hippocampus (***H***; one-way ANOVA: *F*_(2,16)_ = 5.971, *p* = 0.016; 9 controls, 4 demyelinated and 6 remyelinating mice). Data were square root transformed for analysis to satisfy the assumptions of normality and homoscedasticity of the variances. ***I***, ***J***, Quantification of the number of RABV-GFP^+^ neurons within the contralateral cortex of *Pdgfrα-CreER^T2^ :: RphiGT* control, demyelinated, and remyelinating mice (***I***; one-way ANOVA: *F*_(2,16)_ = 8.864, *p* = 0.0026) and the proportion of RABV-GFP^+^ cortical neurons located within the contralateral hemisphere (***J***; one-way ANOVA: *F*_(2,16)_ = 7.49, *p* = 0.005; 9 controls, 4 demyelinated, and 6 remyelinating mice). Data were square root transformed for analysis to satisfy the assumptions of normality and homoscedasticity of the variances. ***K***, ***L***, Quantification of the number of RABV-GFP^+^ neurons within the contralateral hippocampus of *Pdgfrα-CreER^T2^ :: RphiGT* control, demyelinated, and remyelinating mice (***K***; one-way ANOVA: *F*_(2,16)_ = 13.30, *p* = 0.0003) and the proportion of RABV-GFP^+^ hippocampal neurons located within the contralateral hemisphere (***L***; one-way ANOVA: *F*_(2,16)_ = 1.824, *p* = 0.19; 9 controls, 4 demyelinated, and 6 remyelinating mice). Data were square root transformed for analysis to satisfy the assumptions of normality and homoscedasticity of the variances. ***M***, The proportion of RABV-GFP^+^ neurons that were located within the thalamic or hypothalamic nuclei of *Pdgfrα-CreER^T2^ :: RphiGT* control, demyelinated, or remyelinating mice (one-way ANOVA: *F*_(2,16)_ = 0.656, *p* = 0.53). Data were square root transformed for analysis to satisfy the assumptions of normality and homoscedasticity of the variances. ***N***, The proportion of cortical RABV-GFP^+^ neurons located in each cortical region of *Pdgfrα-CreER^T2^ :: RphiGT* control, demyelinated, or remyelinating mice. Note that cortical regions containing >1% of RABV-GFP^+^ neurons are shown. Two-way ANOVA: interaction *F*_(12,112)_ = 2.325, *p* = 0.0108; region *F*_(6,112)_ = 13.75, *p* < 0.0001; treatment *F*_(2,112)_ = 0.00187, *p* = 0.9981. ***O***, The proportion of hippocampal RABV-GFP^+^ neurons within each hippocampal subregion of *Pdgfrα-CreER^T2^ :: RphiGT* control, demyelinated, or remyelinating mice. Two-way ANOVA: interaction *F*_(6,60)_ = 1.042, *p* = 0.4072; region *F*_(3,60)_ = 3,289, *p* < 0.0001, treatment *F*_(2,60)_ = 0.0677, *p* = 0.93047. Data are expressed as mean ± SD. *p ≤ 0.05, **p ≤ 0.01, ****p ≤ 0.0001 by Tukey's multiple-comparison posttest.

To determine whether demyelination or myelin repair alters the type of neuron that synapses with callosal OPCs, we analyzed the location of RABV-GFP^+^ neurons within the brains of control, demyelinated, or remyelinating mice. To induce demyelination, *Pdgfrα-CreER^T2^ :: RphiGT* mice received 5 weeks of cuprizone chow (0.2% w/w), and to induce remyelination, mice were returned to normal chow for a further 5 weeks (Fig. 5*A*). While demyelinated and remyelinating mice had significantly fewer RABV-GFP^+^ neurons per brain compared with control mice (Fig. 4*A*), RABV-GFP^+^ neurons were still primarily located in the ipsilateral hemisphere of control (86 ± 6%), demyelinated (88 ± 3%), and remyelinating (95 ± 4%) mice (Fig. 5*B*,*C*).

Though infected OPCs remained synaptically connected to cortical, hippocampal, and thalamic/hypothalamic neurons following demyelination and remyelination, the relative distribution of the labeled neurons was different in the demyelinated brain. The total number of total cortical RABV-GFP^+^ neurons was unchanged (Fig. 5*E*) but they comprised a greater proportion of the RABV-GFP^+^ neurons in the demyelinated mice (Fig. 5*F*). In the hippocampus, the total number of RABV-GFP^+^ neurons was decreased in demyelinated and remyelinating mice compared with controls, and hippocampal neurons comprised a smaller proportion of total RABV-GFP^+^ neurons (Fig. 5*G*,*H*). When examining the contralateral side of the brain, we determined that the number of RABV-GFP^+^ neurons located within the cortex was decreased in remyelinating mice and represented a smaller proportion of the RABV-GFP^+^ neurons (Fig. 5*I*,*J*). The number of contralateral hippocampal RABV-GFP^+^ neurons was similarly reduced in the remyelinating mice (Fig. 5*K*) but represented a similar proportion of total RABV-GFP^+^ neurons (Fig. 5*L*). The proportion of RABV-GFP^+^ neurons located in the thalamic and hypothalamic nuclei remained low in all conditions, typically accounting for <5% of RABV-GFP^+^ neurons (Fig. 5*M*).

RABV-GFP^+^ neurons were primarily located in the retrosplenial, motor, parietal, somatosensory, visual, auditory, and cingulate cortices of all mice, with a small number (<1% of cortical RABV-GFP^+^ neurons) in the insular, rhinal, limbic, and temporal association cortices (Fig. 5*N*). In remyelinating mice, a higher proportion of RABV-GFP^+^ neurons were found in the retrosplenial cortex and a decreased proportion in the somatosensory cortex compared with controls (Fig. 5*N*), which may suggest that retrosplenial connections are prioritized by OPCs. In contrast, there was no change in the proportion of RABV-GFP^+^ hippocampal neurons located within CA1-3 (Fig. 5*O*).

### OPCs form fewer synaptic connections with layer V pyramidal neuron axons in the demyelinated corpus callosum

**Figure 6.**
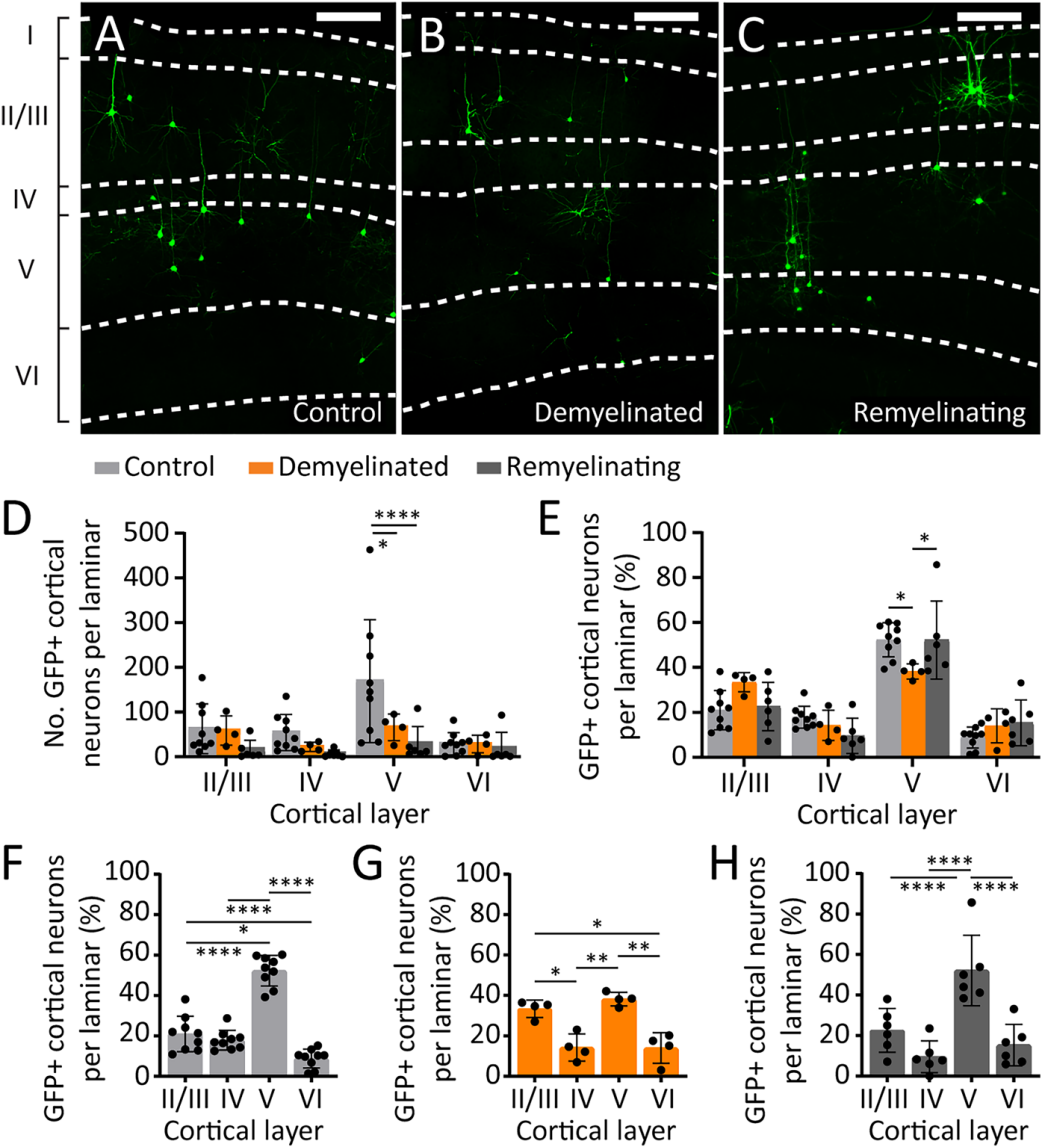
In the demyelinated brain, OPCs receive fewer synapses from layer V and more from layer II/III. ***A***–***C***, Compressed confocal images of the cortex of control (***A***), demyelinated (***B***), and remyelinating (***C***) adult *Pdgfrα-CreER^T2^ :: RphiGT* transgenic mice, 7 d after the SADΔG-GFP-(EnvA) virus was injected into the corpus callosum, showing RABV-GFP^+^ (green) cortical neurons distributed across cortical layers I, II/III, IV, V, and VI. Scale bars represent 100 µm. ***D***, ***E***, The total number (2-way ANOVA: interaction *F*_(6,64)_ = 1.945, *p* = 0.087; laminar *F*_(3,64)_ = 4.196, *p* = 0.009; treatment *F*_(2,64)_ = 8.276, *p* = 0.001) of RABV-GFP^+^ cortical neurons positioned in layers II/III, IV, V, and VI of *Pdgfrα-CreER^T2^:: RphiGT* control, demyelinated, or remyelinating mice (9 controls, 4 demyelinated, and 6 remyelinating mice) and the proportion of RABV-GFP^+^ cortical neurons in each laminar (2-way ANOVA: interaction *F*_(6,64)_ = 3.354, *p* = 0.006; laminar *F*_(3,64)_ = 59.97, *p* < 0.0001; treatment *F*_(2,64)_ = 0.0005, *p* = 0.9995). ***F–H***, Data from ***E***, displaying post hoc analysis of the proportion of RABV-GFP^+^ cortical neurons in cortical laminar II/III, IV, V, and VI of *Pdgfrα-CreER^T2^ :: RphiGT* control (***F***), demyelinated (***G***), or remyelinating (***H***) mice. Data are expressed as mean ± SD. *p ≤ 0.05, **p ≤ 0.01, ***p ≤ 0.001, ****p ≤ 0.0001 by Tukey's multiple-comparisons posttest. Extended Data Figure 6-1 supports Figure 6.

To compare the laminar distribution of RABV-GFP^+^ cortical neurons under control, demyelinated, and remyelinating conditions, RABV-GFP^+^ neurons in the parietal, somatosensory, auditory, and visual cortices were classified as belonging to layer I, II/III, IV, V, or VI (Fig. 6*A–C*). Demyelination was associated with a significant decrease in the number (Fig. 6*D*) and proportion (Fig. 6*E*) of RABV-GFP^+^ cortical neurons located in layer V. Conversely, the proportion of RABV-GFP^+^ cortical neurons located in layer II/III increased in demyelinated mice (*p* = 0.050). In the remyelinating mice, layer V RABV-GFP^+^ cortical neuron number remained low compared with controls (Fig. 6*D*) but comprised a higher proportion of cortical RABV-GFP^+^ neurons than for demyelinated mice (Fig. 6*E*). Indeed, under control conditions, 52 ± 8% of the synapses that OPC formed with cortical neurons were with layer V pyramidal cells and a significantly smaller proportion with layers II/III (21 ± 9%), IV (18 ± 5%), or VI (9 ± 5%; Fig. 6*F*) pyramidal neurons. Following demyelination, the distribution of RABV-GFP^+^ cortical neurons changed, and the proportion of RABV-GFP^+^ neurons was more evenly distributed between layers II/III (33 ± 4%) and V (38 ± 3%; Fig. 6*G*). Following remyelination, the population of cortical neurons that synapsed with callosal OPCs resembled that of control mice, as 52 ± 17% RABV-GFP^+^ neurons were layer V neurons (Fig. 6*H*). These data suggest that following a demyelinating injury, OPCs increasingly synapse with layer II/III and have a similar likelihood of synapsing with layer II/III or layer V pyramidal neurons until myelin is restored, after which they again favor layer V pyramidal cells.

10.1523/ENEURO.0113-25.2025.f6-1 Figure 6-1 **Increased cortical excitatory neuron activity following cuprizone demyelination**. A) Density of NEUN^+^ cells quantified within layers II/II and V of the visual cortex from adult C57bl/6 mice that received 5 weeks of normal chow (control; grey bars) or 0.2% (w/w) cuprizone feed (demyelinated; orange bars) from P67. Two-way ANOVA: region x treatment F(1,8) = 5.508, p=0.046, region F(1,8) = 118.5, p<0.0001, treatment F(1,8) = 29.95, p=0.0006. B) Density of NEUN^+^ CFos^+^ cells within layers II/II and V of the visual cortex of control and demyelinated mice. Two-way ANOVA: region x treatment F(1,8) = 0.3211, p=0.58, region F(1,8) = 1.406, p=0.26, treatment F(1,8) = 34.53, p=0.0004. C) Proportion of NEUN^+^ cells that were CFos^+^ within layers II/II and V of the visual cortex of control and demyelinated mice. Two-way ANOVA: region x treatment F(1,8) = 0.1027, p=0.75, region F(1,8) = 0.077, p=0.78, treatment F(1,8) = 44.62, p=0.0002. D) Corrected total cell fluorescence (CTCF) calculated from CFos^+^ cells within layers II/II and V of the visual cortex of demyelinated mice. Paired t-test: t=3.797, df=2, p=0.0629 *p<0.05, **p<0.01, ****p<0.0001 by Tukey’s post-test. Download Figure 6-1, TIF file.

To explore the possibility that the shift in the population of cortical neurons synapsing with OPCs following demyelination relates to a change in cortical neuron activity, we quantified the expression cFos in neurons within the visual cortex of control and demyelinated mice. Intriguingly, in the visual cortex, cuprizone demyelination reduces the density of NeuN^+^ cells in layer II/III but not layer V (Extended Data Fig. 6-1*A*). The density and proportion of NeuN^+^ cells that express cFos is increased in both layer II/III and layer V in demyelinated mice (Extended Data Fig. 6-1*B,C*) but the relative intensity of cFos expression tends to be greater in cells from layer II/III compared with layer V (Extended Data Fig. 6-1*D*). These data may indicate differential activity changes in cortical neuron populations following demyelination.

## Discussion

OPCs in the developing and adult mouse brain receive synaptic input from neurons (Bergles et al., 2000; Lin and Bergles, 2004; Ziskin et al., 2007). However, it is unclear whether OPCs form synapses with any active axon in their territory or target specific neuron populations. By labeling a subset of OPCs in the adult mouse corpus callosum with a modified rabies virus, which is limited to monosynaptic retrograde tracing, it was possible to visualize neurons that directly synapse onto the labeled OPCs. Infected OPCs received input, almost exclusively, from excitatory cortical and subcortical neurons that project their axons within the vicinity of the infected OPCs. The vast majority of cells labeled by the retrograde virus were ipsilateral CA1 hippocampal neurons and ipsilateral layer V cortical neurons that presumably synapse with the labeled callosal OPCs. Cuprizone demyelination reduced the total number of neurons that synapsed with labeled OPCs but was also associated with callosal OPCs forming synapses equally with layer II/III neurons and layer V cortical neurons. Remyelination was associated with OPCs reverting to the patterns of synaptic communication seen in control mice.

The body of the corpus callosum, at ∼bregma −1.5 mm, primarily contains the axons of neurons from the motor, somatosensory, parietal, and, to a lesser extent, visual, auditory, and retrosplenial cortices (Ku and Torii, 2020). Our data indicate that callosal OPCs synapse with the axons of neurons projecting from each of these regions. As the majority of RABV-GFP^+^ neurons were ipsilateral excitatory pyramidal neurons, OPCs may preferentially synapse with more proximal regions of axons or extend processes to synapse with axons in the external capsule, which contains corticofugal and corticocortical projecting axons, many of which have ipsilateral targets (Khibnik et al., 2014; Allen Mouse Brain Atlas, mouse.brain-map.org/experiment/show/657041814). The large number of ipsilateral RABV-GFP^+^ hippocampal neurons also suggests that OPCs within the CA1 and alveus are synaptically integrated. The alveus is a major efferent tract that allows hippocampal neurons within CA1-3 and the subiculum to send caudal projections into the ipsilateral fimbria-fornix (Daitz and Powell, 1954; Cenquizca and Swanson, 2007; Lingford-Hughes and Kalk, 2012; Duvernoy et al., 2013). RABV-GFP^+^ labeled OPCs may also contact the subset of CA1 hippocampal neurons that project dorsally around the corpus callosum to the retrosplenial, somatosensory, auditory, and visual cortices (Cenquizca and Swanson, 2007) and hippocampal CA1-3 neurons projecting to homotopic and heterotopic contralateral hippocampal regions though the hippocampal commissure (Voneida et al., 1981; Tamamaki et al., 1988; Leung et al., 1992; Cenquizca and Swanson, 2007; Duvernoy et al., 2013). Contact with these hippocampal commissural axons could account for the small number of RABV-GFP^+^ neurons in the contralateral hippocampus.

It was unexpected that ∼52% of RABV-GFP^+^ cortical neurons were layer V neurons and ∼21% were layer II/III neurons as some estimates indicate that layer II/III neurons account for up to 80% of the callosal axon population (Wise, 1975; Fame et al., 2011; Gaspard and Vanderhaeghen, 2011; Chovsepian et al., 2017). These data suggest that in adulthood, OPCs preferentially synapse with layer V axons, an idea that is further supported by OPCs synapsing equally with layer V and layer II/III neurons following demyelination. Layer V can be further subdivided into Va and Vb, and the location of most RABV-GFP^+^ layer V neurons immediately under layer IV (Figs. 2, 6) suggests that they are Va “slender tufted” pyramidal neurons that primarily comprise corticocortical association neurons and callosal commissural neurons (Veinante et al., 2000; Larsen et al., 2007; Groh et al., 2010; Gaspard and Vanderhaeghen, 2011; Grant et al., 2012; Malmierca et al., 2014; Wang et al., 2018).

Layer V pyramidal neurons are among the first population myelinated, with myelin added from ∼P10 in the developing mouse brain (Battefeld et al., 2019) and while less is known about the detailed timing of myelination of layer II/III pyramidal cells, MBP localization suggests that it begins at ∼P21 (Vincze et al., 2008). In the motor cortex, layer V pyramidal neurons receive new myelin internodes throughout life and their myelination can be enhanced by increasing their activity (Gibson et al., 2014). The axons of layer V somatosensory and visual cortical neurons can have continuous stretches of myelination along axons projecting toward the corpus callosum, while layer II/III neurons are intermittently myelinated in adult mice (Tomassy et al., 2014). These observations suggest that layer V neurons are more heavily myelinated than layer II/III neurons. However, the level of myelination can also vary with axon region (Tomassy et al., 2014; Call and Bergles, 2021) and only ∼8% of the somatosensory layer V axon collaterals that project to layer I are myelinated, and of those, most are sparsely myelinated (Call and Bergles, 2021). It is unclear whether layer V axons that traverse the corpus callosum are heavily or sparsely myelinated. However, as OPCs form synapses with unmyelinated axons segments (Kukley et al., 2007; Ziskin et al., 2007; Gautier et al., 2015), our data suggest that layer V axons are intermittently myelinated in the corpus callosum.

Intrinsic neuronal properties impact neuronal activity and influence the formation of axon→OPC synapses. Under normal conditions, layers II/III and Va pyramidal neurons have similar firing patterns, both generating trains of regularly spaced, or intermittent but repetitive action potential bursts upon depolarization (Dégenètais et al., 2002; Moyer et al., 2002). As OPCs preferentially synapse with electrically active neurons (Moura et al., 2023), it is possible that OPCs target layer II/III neurons in the demyelinated corpus callosum because of their altered firing pattern. Whole-cell patch-clamp recordings from layer II/III neurons in the demyelinated anterior cingulate cortex indicate that more current is required to elicit an equivalent action potential firing frequency (Kawabata et al., 2025). However, this may be a homeostatic plasticity response to reduce firing, as cuprizone demyelination is associated with a significant increase in the proportion of layers II/III and V neurons that express cFos in the visual cortex (Extended Data Fig. 6-1). A detailed study of spontaneous activity across the cortical laminar would be required to understand whether the relative activity of these populations might explain the change in OPC preference following demyelination.

We predict that OPC synapse formation precedes differentiation and remyelination. High-frequency action potential trains can increase the amount of free residual calcium within axons and facilitate glutamate release at axon→OPC synapses (Nagy et al., 2017). In vivo, the high-frequency stimulation of axons in the corpus callosum of adult mice is also associated with increased OPC differentiation and an increase in the number of newborn EdU^+^ oligodendrocytes (Nagy et al., 2017). Similarly, the optogenetic or chemogenetic activation of a subset of cortical neurons promotes the remyelination of these neurons in the corpus callosum of healthy (Gibson et al., 2014; Mitew et al., 2018) and demyelinated mice (Ortiz et al., 2019). Given the high proportion of callosal axons that are remyelinated after cuprizone withdrawal, this is likely to include a substantial number of layer II/III neurons. It would be interesting to determine the increase in layer II/III neuron→OPCs synapse formation indicate that layer II/III neurons are prioritized for remyelination.

Our study provides further evidence that OPCs located in the corpus callosum preferentially form synaptic connections with neuronal subpopulations predominantly in the cortex and hippocampus. By showing that OPCs can synapse with different neurons in health and following demyelination, we provide further insight into neuron-OPC communication and the cellular interactions that are impacted in demyelinating diseases such as multiple sclerosis.

## Data Availability

All individual data points are provided in the data figures or in the extended data of the manuscript. Requests for any other data files should be directed to Dr. Carlie Cullen (carlie.cullen@mater.uq.edu.au) or Prof. Kaylene Young (kaylene.young@utas.edu.au).

## Synthesis

Reviewing Editor: Benjamin Deneen, Baylor College of Medicine Decisions are customarily a result of the Reviewing Editor and the peer reviewers coming together and discussing their recommendations until a consensus is reached. When revisions are invited, a fact-based synthesis statement explaining their decision and outlining what is needed to prepare a revision will be listed below. The following reviewer(s) agreed to reveal their identity: erin gibson.

The reviewers hold a generally positive view of this manuscript. They have suggested a series of revisions that will significantly improve the manuscript. Accordingly, as you revise this manuscript, please take these suggestions under advisement.

Reviewer 1

The authors use a previously established retrograde monosynaptic labelling strategy in mice to characterise the extent of synaptic input of different neuronal populations onto oligodendrocyte precursor cells (OPCs) in the corpus callosum. They show that in heathly mice, the majority of callosal neuron-to-OPC synapses are formed by layer 5 cortical neurons, and to a lesser extent by layer 2/3 neurons and hippocamapal neurons. However, following Cuprizone-mediated demyelination, the authors reveal a shift toward increased synaptic connectivity between OPCs and layer 2/3 neurons while total synaptic input remains unchanged. The authors conclude from these data that demyelinated axons compete for synaptic connections with OPCs.

The manuscript is well written and easy to follow. The presentation of the data is of high quality and transparent. I don't have much to comment here, if anything.

The observations that the authors present here are intriguing and very interesting as they show that OPCs are not randomly integrated into neuronal networks in the healthy CNS, and that this synaptic integration shifts between neuronal populations in demyelination. It would of course be very interesting to understand better what this shift means for OPC behaviour as well as to why this shift occurs, and how OPCs are differently connected to these differentially myelinated groups of neurons in the first place. However, I understand that these questions are beyond the scope of a revision and I regard the results as interesting and publication-worthy as they are.

I have a few minor comments to take into consideration:

- the authors provide an interesting discussion in stating that the majority of callosal axons are from layer 2/3 neurons, but that these only represent a minority of synaptically connected ones (opposite to layer 5 neurons). Furthermore, it is the layer 5 neurons which are the most robustly myelinated ones in terms of frequency of myelination as well as extent of myelin coverage along the length of the axon. Where do the authors think OPC synapses might be formed along axons of layer 5 neurons and what could be their purpose if they already have a lot of myelin (i.e. there isn't much space where a axon:OPC synapse could be, opposite to layer 2/3 neurons)? I would be interesting to discuss this a little further.

- Which axons are preferentially remyelinated following Cuprizone? Is it the previously myelinated layer 5 neurons or is it the layer 2/3 neurons? Knowledge or discussion of these events would help draw further scenarios on the roles that OPC synapses might have with regard to myelination or myelination-independent functions on these different neuronal populations.

- The title states "Demyelinated cortical neurons compete to form synaptic connections...". The term "to compete" is an interpretation that is not substantiated by the data shown, and thus an overstatement. I would suggest to change the title to reflect more appropriately what the authors show, namely a shift between neuronal populations.

Reviewer 2

Comments to the Authors (Required) This paper investigated how neuron-OPC synaptic connectivity is altered in the context of cuprizone-driven demyelinating. Using retrograde rabies tracing targeted to corpus callosal OPCs, the authors found that while the number of synapses onto the OPCs remains consistent, the ratio of cortical layer V to layer II/III labeled shifts in favor of layer II/III neurons. The authors provide a thorough characterization of the viral technique before and during demyelination. They find that labeled OPCs only survive for a few days, but that this is sufficient to infect upstream neurons. The majority of the labeled OPCs are found in the corpus callosum, though some are found within the cortex and hippocampus. Interestingly, the virus appeared to spread more throughout the CC in the demyelinated brains, which the authors attribute to a decrease in CC density. Finally, the authors investigated N-O connectivity through patch clamp recordings of mEPSCs and concluded that there was no change in connectivity in response to cuprizone demyelination. Overall, this paper appears rigorous and begins to touch upon the question of how oligodendroglia dynamically choose which neurons to myelinate. While the conclusions of this paper address N-O synaptic connectivity, it does not do not directly answer the question of how this would affect myelination. However, it is informative and an important step for the field as we work towards this question.

Here are some concerns and suggestions that would help clarify and strengthen the paper:

1) I would highly suggest supplementary figures and/or table with OPC resting resistance, capacitance, and potential as these are informative to understand the quality of the recording data but also understand how OPCs are changing in response to cuprizone. Changes in resistance, for example, will alter the amplitudes of mEPSCs and may be confounding for the conclusions on N-O connectivity made from the data in Figure 4 I.

2) A major concern is centered around the conclusions about hippocampal effects. Throughout the text, the authors acknowledge that the viral injection reaches the hippocampus. Therefore, any neuron that is labeled in green is likely due to the technical confound of infected OPCs within the hippocampus, and potentially not from callosal OPCs. The authors' reasoning on how hippocampal neurons were labeled by callosal OPCs is highly speculative and a stretch, especially due to the lack of previous literature showing callosal projections by hippocampal neurons, and the simpler explanation of hippocampal OPCs being infected. If the authors feel strongly about the claim that callosal OPCs are connected to hippocampal neurons, then stronger evidence needs to be provided. For example, citing literature, anterograde labeling of hippocampal neurons, recordings in callosal OPCs with hippocampal stimulation, and dual patching of hippocampal neurons and callosal OPCs. The authors' data show that hippocampal neuron connectivity to callosal OPCs is unaffected by the cuprizone paradigm. The lack of effect highlights that the two or likely orthogonal since it is unlikely that they are functionally connected by synapses. I suggest noting that OPCs and neurons in the hippocampus are labeled for transparency and accuracy but omitting any conclusions about the hippocampus due to the technical confound and lack of precident and current evidence. Conclusions about hippocampus can be distracting from the more convincing cortical results.

3) I would strongly suggest including figure panels outlining when cuprizone and tamoxifen were administered, and when animals were sacrificed. This will help with clarity and consistency of information. Could the authors provide more justification on why tamoxifen was administered at different times as the time windows between tamoxifen injection, cuprizone administration, and viral infections are not consistent between paradigms and is an important confound to note for the readers.

4) "The number and location of RABV-GFP+ neurons was consistent for the healthy mice analysed previously (see Fig. 2) and the control mice analysed for this study (statistical comparison in supplementary table 1)." Referencing the figures is confusing as the figures for the control mice are not referenced. Are the data in figure 5?

5) I agree with the authors usage of the capacitance and size of sodium currents as selection criteria for OPCs. However, as sodium current amplitude is dependent on voltage and step size, can the authors be more specific on when the 100 pA currents needed to be observed to fit the criteria?

6) Can the authors give more motivation on why RuR was used? What information does it add in this context? I imagine this might be because mEPSC frequency is low at baseline in OPCs, but explicit motivation is needed in the text.

7) More interpretation of the mEPSC data is needed for them to be relevant for the story. For example, changes in amplitude and decay times are likely driven by post-synaptic effects of receptor expression and localization instead of presynaptic changes. Since the context of the experiments is to study the connectivity changes, these measurements do not seem to support the question asked by the authors, though they are possibly interesting. There is a possibility that amplitude or decay could be presynaptic if layer II/III neurons release different amounts of neurotransmitter than layer V neurons, but as the general thought is that neurotransmitters are released at saturating levels, it could be hard to detect in these experiments. Overall, more context and interpretation by the authors is needed to understand how these experiments are relevant to the story.

8) "It is possible that the OPCs target layer II/III neurons in the demyelinated brain because they become hyperactive." Is there literature to support specific hyperactivity of layer II/III neurons compared to layer V? Or do authors have data that could support this claim?

9) Figure S1. Could panel letters be added in the text for ease of the reader, please? And generally, could the use of red and green in the same panels be avoided to ensure colour blind readers can easily visualize the data? A common alternative is magenta instead of red.

10) Notes on statistics: I appreciate the authors transparency and detail on the statistics used. I also believe that the experimental design was rigorous allowing for meaningful statistics to be performed. However, below are some critiques about the statistics.

• I agree with the authors' choice to check for normality of the data, however I would generally avoid non-parametric tests as they still require the underlying assumptions of equal shape and spread (which can be caused by non-normality of the data and therefore non-parametric tests do not address the original issue of normality), and are generally less powerful once normality of the data and variance are met. Transformations such as Log10 (as mentioned by the authors), square root for count data (eg. neuron count), or Logit for percentages, can be used to address heteroscedasticity and normality of variance.

• An example of transforming data to use a parametric test is with the data in Fig 4a, where the authors note: "The number of RABV-GFP+ neurons appeared higher in control than demyelinated mice (Figure 4A), but this did not reach statistical significance." The count data can be square-rooted, which will normalize the spread and allow for the use of parametric tests. I attempted to model the data in Fig 4a with the transformation and found that using a GLM standard least squares produces a statistically significant model, and a subsequent multiple comparisons (eg. Tukey) will yeild a significant result for C vs D and C vs R. This supports the authors' assessment that the groups were different than control.

• In order to ensure independence of measurements, a blocking factor of cage could be included in the statistical model. This is because the drug is applied at the level of cage, and so animals within the cage are not independent. This will mitigate pseudo-replication and may lead to increased statistical significance as the model can account for within-cage variance.

• There is a general issue of repeated measures in data within figures 4I-J as they are drawn from some events that happen within the same cell and therefore are not independent measurements and pseudo-replicated. Calculating the area under the curve (AUC) and performing statistical test on that single value can circumvent this issue.

• Figure 4E-H: The data presented are cells. To avoid pseudo-replication, the statistical model must also include the animal as the unit.

## Author Response

Reply to reviewers re: Manuscript eN-NWR-0113-25 We thank the reviewers for their time and effort reviewing our paper and feel their constructive and considered feedback have improved our manuscript. Herein we include our detailed response to each reviewer comment.

Reviewer 1: Where do the authors think OPC synapses might be formed along axons of layer 5 neurons and what could be their purpose if they already have a lot of myelin (i.e. there isn't much space where a axon:OPC synapse could be, opposite to layer 2/3 neurons)? I would be interesting to discuss this a little further.

We agree that this is an interesting point to explore further and have added the following paragraph to the discussion on page 18 of the manuscript. 'Layer V pyramidal neurons are amongst the first population myelinated, with myelin added from ~P10 in the developing mouse brain (Battefeld et al., 2019) and while less is known about the detailed timing of myelination of layer II/III pyramidal cells, MBP localisation suggests that it begins at ~P21 (Vincze et al., 2008). In the motor cortex, layer V pyramidal neurons receive new myelin internodes throughout life and their myelination can be enhanced by increasing their activity (Gibson et al., 2014). The axons of layer V somatosensory and visual cortical neurons can have continuous stretches of myelination along axons projecting towards the corpus callosum, while layer II/III neurons are intermittently myelinated in adult mice (Tomassy et al., 2014). These observations suggest that layer V neurons are more heavily myelinated than layer II/III neurons. However, the level of myelination can also vary with axon region (Tomassy et al., 2014; Call and Bergles, 2021) and only ~8% of the somatosensory layer V axon collaterals that project to layer I are myelinated, and of those, most are sparsely myelinated (Call and Bergles, 2021). It is unclear whether layer V axons that traverse the corpus callosum are heavily or sparsely myelinated. However, as OPCs form synapses with unmyelinated axons segments (Kukley et al., 2007; Ziskin et al., 2007; Gautier et al., 2015), our data suggest that layer V axons are intermittently myelinated in the corpus callosum.' Reviewer 1: Which axons are preferentially remyelinated following Cuprizone? Is it the previously myelinated layer 5 neurons or is it the layer 2/3 neurons? Knowledge or discussion of these events would help draw further scenarios on the roles that OPC synapses might have with regard to myelination or myelination-independent functions on these different neuronal populations.

In our current study we were unable to determine which cortical neuron populations were preferentially remyelinated. However we have discussed the possibility that layer II/III neurons are preferentially remyelinated and why this might be the case the following paragraphs which can be found on pages 18-19 our revised manuscript. 'Intrinsic neuronal properties impact neuronal activity and influence the formation of axon-OPC synapses. Under normal conditions, layers II/III and Va pyramidal neurons have similar firing patterns, both generating trains of regularly spaced, or intermittent but repetitive action potential bursts upon depolarisation (Dégenètais et al., 2002; Moyer et al., 2002). As OPCs preferentially synapse with electrically active neurons (Moura et al., 2023), it is possible that OPCs target layer II/III neurons in the demyelinated corpus callosum because of their altered firing pattern. Whole cell patch clamp recordings from layer II/III neurons in the demyelinated anterior cingulate cortex indicate that more current is required to elicit an equivalent action potential firing frequency (Kawabata et al., 2025). However, this may be a homeostatic plasticity response to reduce firing, as cuprizone demyelination is associated with a significant increase in the proportion of layers II/III and V neurons that express cFos in the visual cortex (Figure 6-1). A detailed study of spontaneous activity across the cortical laminar would be required to understand whether the relative activity of these populations might explain the change in OPC preference following demyelination.

We predict that OPC synapse formation precedes differentiation and remyelination. High frequency action potential trains can increase the amount of free residual calcium within axons and facilitate glutamate release at axon-OPC synapses (Nagy et al., 2017). In vivo, the high frequency stimulation of axons in the corpus callosum of adult mice is also associated with increased OPC differentiation and an increase in the number of newborn EdU+ oligodendrocytes (Nagy et al., 2017). Similarly, the optogenetic or chemogenetic activation of a subset of cortical neurons promotes the remyelination of these neurons in the corpus callosum of healthy (Gibson et al., 2014; Mitew et al., 2018) and demyelinated mice (Ortiz et al., 2019). Given the high proportion of callosal axons that are remyelinated after cuprizone withdrawal, this is likely to include a substantial number of layer II/III neurons. It would be interesting to determine the increase in layer II/III neuron-OPCs synapse formation indicate that layer II/III neurons are prioritised for remyelination. ' Reviewer 1: The title states "Demyelinated cortical neurons compete to form synaptic connections...". The term "to compete" is an interpretation that is not substantiated by the data shown, and thus an overstatement. I would suggest to change the title to reflect more appropriately what the authors show, namely a shift between neuronal populations.

We have now changed the paper title to "Demyelination produces a shift in the population of cortical neurons that synapse with callosal oligodendrocyte progenitor cells".

Reviewer 2: I would highly suggest supplementary figures and/or table with OPC resting resistance, capacitance, and potential as these are informative to understand the quality of the recording data but also understand how OPCs are changing in response to cuprizone.

We now include Table 3 detailing the basic membrane properties of patched OPCs.

Reviewer 2: A major concern is centered around the conclusions about hippocampal effects. Throughout the text, the authors acknowledge that the viral injection reaches the hippocampus. Therefore, any neuron that is labeled in green is likely due to the technical confound of infected OPCs within the hippocampus, and potentially not from callosal OPCs. The authors' reasoning on how hippocampal neurons were labeled by callosal OPCs is highly speculative and a stretch, especially due to the lack of previous literature showing callosal projections by hippocampal neurons, and the simpler explanation of hippocampal OPCs being infected.... I suggest noting that OPCs and neurons in the hippocampus are labeled for transparency and accuracy but omitting any conclusions about the hippocampus due to the technical confound and lack of precident and current evidence. Conclusions about hippocampus can be distracting from the more convincing cortical results.

The reviewer makes an extremely valid point. We have gone through the manuscript to carefully remove / edit any text that implied the callosal OPCs were connected to hippocampal axons within the corpus callosum and clarified OPC infection within the alveus and CA1 as the likely source of hippocampal neuron labelling.

Reviewer 2: I would strongly suggest including figure panels outlining when cuprizone and tamoxifen were administered, and when animals were sacrificed. This will help with clarity and consistency of information. Could the authors provide more justification on why tamoxifen was administered at different times as the time windows between tamoxifen injection, cuprizone administration, and viral infections are not consistent between paradigms and is an important confound to note for the readers.

We now include experimental time-course schematics in the figure panels.

We have also added additional justification of experimental design to the methods section of the manuscript: "Mice received 4 consecutive doses of Tamoxifen, as we have previously shown that this dosing regimen reliably activates Cre recombinase in essentially all OPCs in Pdgfra- CreERTM mice (REFS). We also ensured a minimum of 3 days between the final tamoxifen dose and commencing any intervention, to ensure that OPC recombination was complete and stable prior to viral injection. We avoided the simultaneous delivery of tamoxifen and cuprizone, as this can result in weight loss, but primarily because tamoxifen can promote remyelination (REFS). To evaluate neuron-OPC synaptic connectivity in remyelinating mice, tamoxifen delivery was delayed until the end of the remyelinating period, to ensure that essentially all callosal OPCs, including those generated from neural stem cells over the 10-week tracing period, were susceptible to viral infection." Reviewer 2: "The number and location of RABV-GFP+ neurons was consistent for the healthy mice analysed previously (see Fig. 2) and the control mice analysed for this study (statistical comparison in supplementary table 1)." Referencing the figures is confusing as the figures for the control mice are not referenced.

We have modified the text on page 14 to improve clarity. "To determine if the population of neurons that forms synaptic connections with callosal OPCs was altered by demyelination, Pdgfrα-CreERT2 :: RphiGT mice received either 5-weeks of standard or cuprizone chow (0.2% w/w) prior to SADΔG-GFP-(EnvA) viral injection at P85. Mice were then maintained on standard chow for 7-days to ensure robust neuronal RABV-GFP expression. We first compared the number and location of RABV-GFP+ neurons in the P85+7D control mice (n=5) with data obtained from our previous P49+7D control group (n=4; Fig. 2) and found that data from these control groups were equivalent (statistical comparison in Table 1). Therefore, data from n=9 control mice were combined to determine whether demyelination altered RABV-GFP neuronal labelling." Reviewer 2: I agree with the authors usage of the capacitance and size of sodium currents as selection criteria for OPCs. However, as sodium current amplitude is dependent on voltage and step size, can the authors be more specific on when the 100 pA currents needed to be observed to fit the criteria? Additional text has been added to page 5 to clarify the methodology. "Upon breakthrough, resting membrane potential (RMP), capacitance, membrane resistance, and the magnitude of the voltage-gated inward (sodium) current were recorded using a HEKA patch clamp EPC800 amplifier and pclamp 10.5 software (Molecular devices) as previously described (Pitman et al., 2020). The voltage-gated inward current was elicited by a voltage step from −70 mV to 0 mV, as OPCs produce a transient inward current after depolarization beyond −30 mV, and cells with a NaV conductance {greater than or equal to} 100 pA and capacitance (Cm) of <50 pF were classified as OPCs (as per Clarke et al., 2012; Ferreira et al., 2020)." Reviewer 2: Can the authors give more motivation on why RuR was used? What information does it add in this context? I imagine this might be because mEPSC frequency is low at baseline in OPCs, but explicit motivation is needed in the text.

We have now added a sentence to the results text on page 14 indicating that: "We would not expect a change in the composition of presynaptic neurons to impact mEPSC properties. This was confirmed by measurement of mEPSC amplitude and decay time (Figure 4F-G). However, the low frequency of mEPSCs in OPCs restricted the number of events available for analysis. To overcome this limitation, OPCs were exposed to the secretagogue, ruthenium red, to increase the frequency of glutamate release in the presence of TTX. This increased mEPSC frequency significantly for control and demyelinated OPCs (Figure 4H) and increased the number of mEPSCs that could be evaluated to confirm that mEPSC amplitude (Figure 4I) and decay time (Figure 4J) were unchanged by demyelination." Reviewer 2: More interpretation of the mEPSC data is needed for them to be relevant for the story. For example, changes in amplitude and decay times are likely driven by post-synaptic effects of receptor expression and localization instead of presynaptic changes. Since the context of the experiments is to study the connectivity changes, these measurements do not seem to support the question asked by the authors, though they are possibly interesting. There is a possibility that amplitude or decay could be presynaptic if layer II/III neurons release different amounts of neurotransmitter than layer V neurons, but as the general thought is that neurotransmitters are released at saturating levels, it could be hard to detect in these experiments. Overall, more context and interpretation by the authors is needed to understand how these experiments are relevant to the story.

Please see reply to the previous comment, as our edits to explain the use of ruthenium red encompasses this explanation.

Reviewer 2: "It is possible that the OPCs target layer II/III neurons in the demyelinated brain because they become hyperactive." Is there literature to support specific hyperactivity of layer II/III neurons compared to layer V? Or do authors have data that could support this claim? We now include additional extended data figure Figure 6-1 quantifying cFos in neun+ cells in layers II/III and V of the control and demyelinated visual cortex.

We also provide additional discussion of the relative activity changes in these populations on pages 18-19 (see response to reviewer 1).

Reviewer 2: Figure S1. Could panel letters be added in the text for ease of the reader, please? And generally, could the use of red and green in the same panels be avoided to ensure colour blind readers can easily visualize the data? A common alternative is magenta instead of red.

We have added panels letters as requested for Figure S1. We have also changed the red to magenta. This Figure is now Figure 1-1 in accordance with journal policy.

Reviewer 2: I agree with the authors' choice to check for normality of the data, however I would generally avoid non-parametric tests as they still require the underlying assumptions of equal shape and spread (which can be caused by non-normality of the data and therefore non-parametric tests do not address the original issue of normality), and are generally less powerful once normality of the data and variance are met. Transformations such as Log10, square root, or Logit, can be used to address heteroscedasticity and normality of variance.

We have transformed our data to reduce the use of non-parametric tests, and included the following statement in the statistical methods on page 9. "Data that were not normally distributed were square root transformed to satisfy assumptions of normality and homoscedasticity of the variances followed by analysis by parametric tests where indicated" • An example of transforming data to use a parametric test is with the data in Fig 4a, where the authors note: "The number of RABV-GFP+ neurons appeared higher in control than demyelinated mice (Figure 4A), but this did not reach statistical significance." The count data can be square-rooted, which will normalize the spread and allow for the use of parametric tests. I attempted to model the data in Fig 4a with the transformation and found that using a GLM standard least squares produces a statistically significant model, and a subsequent multiple comparisons (eg. Tukey) will yeild a significant result for C vs D and C vs R. This supports the authors' assessment that the groups were different than control.

We have taken the reviewer's advice and square root transformed out data sets and have changed the text, figures and legends accordingly. For example, on page 14: "The number of RABV-GFP+ neurons labelled in control mice was highly variable (F test to compare variance, p=0.0083), however, a square root data transformation prior to data analysis, revealed that fewer RABV-GFP+ neurons were labelled per mouse in demyelinated mice (Fig. 4A)." Reviewer 2: In order to ensure independence of measurements, a blocking factor of cage could be included in the statistical model. This is because the drug is applied at the level of cage, and so animals within the cage are not independent. This will mitigate pseudo-replication and may lead to increased statistical significance as the model can account for within-cage variance.

We appreciate the reviewer's suggestion. This would certainly be appropriate for some experimental designs but is not a major factor for this study. We have gone back to the methods to ensure that we explain that the cuprizone chow is made up every second day as a single experimental batch and is then distributed across the different cages. The chow is not made up separately for each cage. If we could measure the dietary intake of individual mice within each cage, that would certainly be an impactful variable, but this is not something we can measure.

Reviewer 2: There is a general issue of repeated measures in data within figures 4I-J as they are drawn from some events that happen within the same cell and therefore are not independent measurements and pseudo-replicated. Calculating the area under the curve (AUC) and performing statistical test on that single value can circumvent this issue.

We have revisited data presented in Figures 4I and 4J and agree that this was pseudo replication and not informative. Therefore, we have removed these data panels from the manuscript. • Figure 4E-H: The data presented are cells. To avoid pseudo-replication, the statistical model must also include the animal as the unit.

Data presented in Figure 4E-H and new Figure 4I-J are from individual cells, however, we now perform a REML and have included mice as a random effect term. We have also updated our statistical methods text on page 9 to reflect this. "mEPSC data were analysed in R (v4.4.2) using lme4, emmeans, and easystats packages for restriction maximum likelihood (REML) linear mixed models to determine the effect of cuprizone feeding with individual mouse included as a random effect term."
