# Supplementary figures and images for: Demyelination Produces a Shift in the Population of Cortical Neurons That Synapse with Callosal Oligodendrocyte Progenitor Cells
Source: eNeuro. 2025 Jun 12;12(6):ENEURO.0113-25.2025. doi: 10.1523/ENEURO.0113-25.2025 (PMC12177707; doi:10.1523/ENEURO.0113-25.2025)

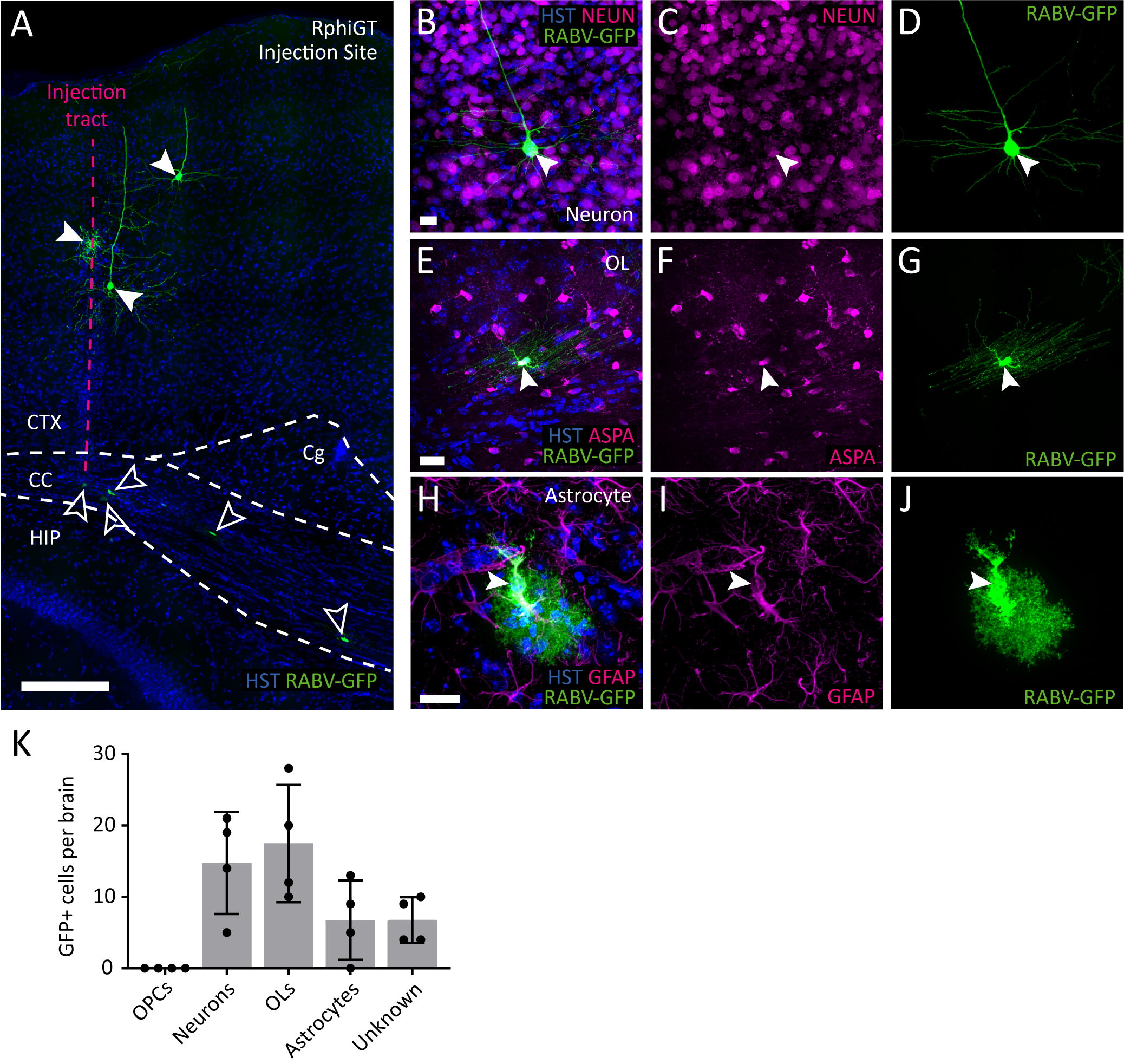

Supplement: Figure 1-1 — Non-specific uptake of the SADΔG-GFP-(EnvA) virus in vivo (A) Compressed confocal image of a coronal brain section from a P42+14 RphiGT control mouse 7-days post-injection of the SADΔG-GFP-(EnvA) virus (green) into the corpus callosum (CC), with Hoechst 33342 nuclear stain (HST, blue). The microinjection tract (red dashed line), cortex (CTX), CC, cingulum (Cg), and hippocampus (HIP) are indicated on the image. Solid arrows highlight RABV-GFP+ cortical neurons. Hollow arrows highlight RABV-GFP+ OLs. (B-D) Compressed confocal image showing a RABV-GFP+ (green) NEUN+ (red) cortical pyramidal neuron, with a HST+ nucleus (blue). (E-G) Compressed confocal image showing a RABV-GFP+ (green) ASPA+ (red) callosal OL, with a HST+ nucleus (blue). (H-J) Compressed confocal image showing a RABV-GFP+ (green) GFAP+ (red) cortical astrocyte, with a HST+ nucleus (blue). (K) Quantification of the total number of RABV-GFP+ OPCs, neurons, OLs, astrocytes, and unidentified cells per P42+14 RphiGT (n = 4) control mouse (mean ± SD). Scale bars represent 200 µm (A) or 20 µm (B-H). Download Figure 1-1, TIF file. [file eneuro-12-ENEURO.0113-25.2025-s003.tif]

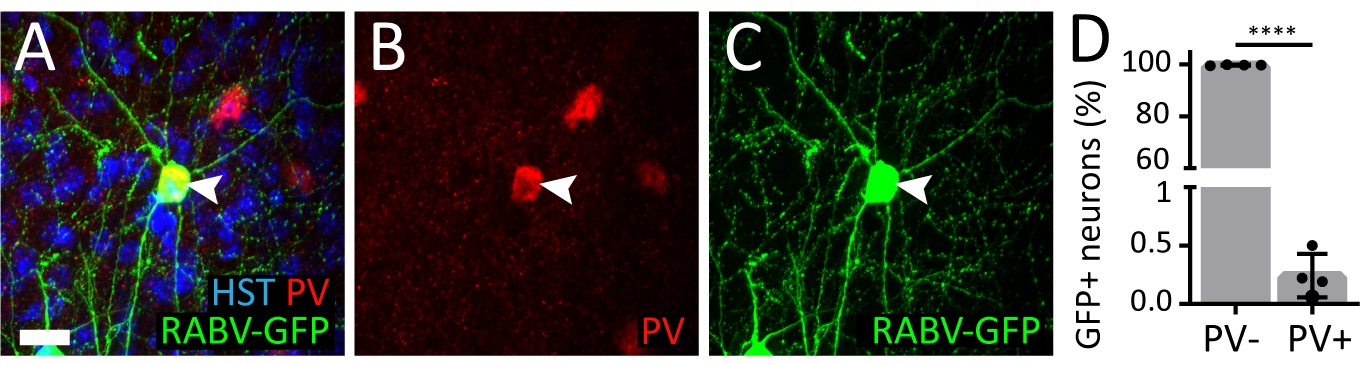

Supplement: Figure 2-1 — Callosal OPCs receive minimal synaptic input from parvalbumin interneurons (A-C) Representative compressed confocal image of a RABV-GFP+ (green), parvalbumin+ (PV; red) interneuron, with a Hoechst 33342 (HST) labelled nucleus (blue) in the cortex of a P42+14 Pdgfrα-CreERT2 :: RphiGT transgenic mouse 7-days after SADΔG-GFP-(EnvA) virus was injected into the corpus callosum. Scale bar represents 20 μm. (D) The proportion of RABV-GFP+ neurons that co-label for PV in Pdgfrα-CreERT2 :: RphiGT transgenic mice (n = 4; mean ± SD). Paired t-test; t (3) = 537.4, p<0.0001. ****p ≤ 0.0001. Download Figure 2-1, TIF file. [file eneuro-12-ENEURO.0113-25.2025-s004.tif]

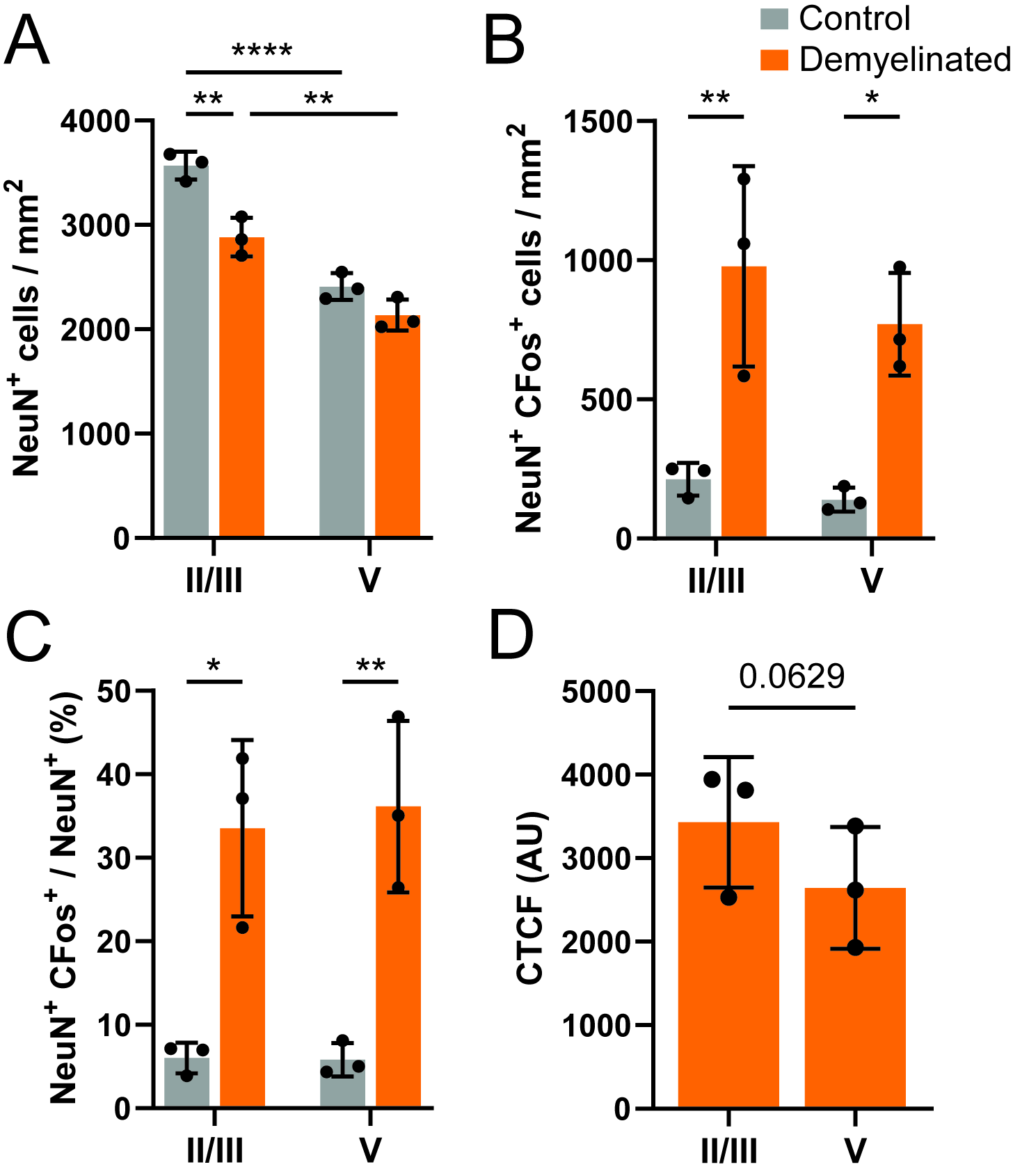

Supplement: Figure 6-1 — Increased cortical excitatory neuron activity following cuprizone demyelination. A) Density of NEUN+ cells quantified within layers II/II and V of the visual cortex from adult C57bl/6 mice that received 5 weeks of normal chow (control; grey bars) or 0.2% (w/w) cuprizone feed (demyelinated; orange bars) from P67. Two-way ANOVA: region x treatment F(1,8) = 5.508, p=0.046, region F(1,8) = 118.5, p<0.0001, treatment F(1,8) = 29.95, p=0.0006. B) Density of NEUN+ CFos+ cells within layers II/II and V of the visual cortex of control and demyelinated mice. Two-way ANOVA: region x treatment F(1,8) = 0.3211, p=0.58, region F(1,8) = 1.406, p=0.26, treatment F(1,8) = 34.53, p=0.0004. C) Proportion of NEUN+ cells that were CFos+ within layers II/II and V of the visual cortex of control and demyelinated mice. Two-way ANOVA: region x treatment F(1,8) = 0.1027, p=0.75, region F(1,8) = 0.077, p=0.78, treatment F(1,8) = 44.62, p=0.0002. D) Corrected total cell fluorescence (CTCF) calculated from CFos+ cells within layers II/II and V of the visual cortex of demyelinated mice. Paired t-test: t=3.797, df=2, p=0.0629 *p<0.05, **p<0.01, ****p<0.0001 by Tukey’s post-test. Download Figure 6-1, TIF file. [file eneuro-12-ENEURO.0113-25.2025-s005.tif]
